# Expression of Functional Human Sialyltransferases ST3Gal1 and ST6Gal1 in *Escherichia coli*

**DOI:** 10.1371/journal.pone.0155410

**Published:** 2016-05-11

**Authors:** Maria Elena Ortiz-Soto, Jürgen Seibel

**Affiliations:** Institut für Organische Chemie, Julius-Maximilians-Universität, Am Hubland, 97074, Würzburg, Germany; University Paris Diderot-Paris 7, FRANCE

## Abstract

Sialyltransferases (STs) are disulfide-containing, type II transmembrane glycoproteins that catalyze the transfer of sialic acid to proteins and lipids and participate in the synthesis of the core structure oligosaccharides of human milk. Sialic acids are found at the outermost position of glycostructures, playing a key role in health and disease. Sialylation is also essential for the production of recombinant therapeutic proteins (RTPs). Despite their importance, availability of sialyltransferases is limited due to the low levels of stable, soluble and active protein produced in bacterial expression systems, which hampers biochemical and structural studies on these enzymes and restricts biotechnological applications. We report the successful expression of active human sialyltransferases ST3Gal1 and ST6Gal1 in commercial *Escherichia coli* strains designed for production of disulfide-containing proteins. Fusion of hST3Gal1 with different solubility enhancers and substitution of exposed hydrophobic amino acids by negatively charged residues (supercharging-like approach) were performed to promote solubility and folding. Co-expression of sialyltransferases with the chaperon/foldases sulfhydryl oxidase, protein disulfide isomerase and disulfide isomerase C was explored to improve the formation of native disulfide bonds. Active sialyltransferases fused with maltose binding protein (MBP) were obtained in sufficient amounts for biochemical and structural studies when expressed under oxidative conditions and co-expression of folding factors increased the yields of active and properly folded sialyltransferases by 20%. Mutation of exposed hydrophobic amino acids increased recovery of active enzyme by 2.5-fold, yielding about 7 mg of purified protein per liter culture. Functionality of recombinant enzymes was evaluated in the synthesis of sialosides from the β-d-galactoside substrates lactose, *N*-acetyllactosamine and benzyl 2-acetamido-2-deoxy-3-*O*-(β-d-galactopyranosyl)-α-d-galactopyranoside.

## Introduction

Biosynthesis of glycoproteins and glycolipids in eukaryotes is performed by a combined and ordered sequential action of glycosidases and glycosyltransferases mainly in the rough endoplasmic reticulum (ER) and Golgi apparatus.[1] Complex carbohydrates display fucose and sialic acids at terminal positions. Given their outermost position on glycoconjugates, sialic acids play a key role in many physiological and pathological events, thus an altered sialylation pattern is often associated with disease.[2] For example, altered sialylation is a hallmark of cancer and overexpressed sialylated glycans are cancer biomarkers.[2] Native sialylation is also critical for the function of therapeutic proteins since it affects physical, chemical and immunogenic properties of glycoproteins.[3] Sialyltransferases are responsible for the transfer of sialic acids from CMP-sialic acid onto either a terminal galactose, *N*-acetylgalactosamine or other sialic acid linked to glycoproteins or glycolipids resulting in α2–3, α2–6 and α2–8 linkages. Consequently, production of large amounts of catalytically active STs is of interest for biotechnological applications, including development of STs inhibitors for cancer therapy and *in vitro* sialylation of TRPs.[4, 5]

Based on their regioselectivity and according to their acceptor specificity mammalian sialyltransferases (Glycosyltransferase family 29 according to CAZy classification) are grouped in four subfamilies: ST3Gal (I-VI), ST6Gal (I and II), ST6GalNAc (I-VI) and ST8Sia (I-IV).[6, 7] Members within each subfamily show conserved cysteine residues involved in the formation of disulfide bonds that are important for proper protein folding and activity.[8, 9] Human STs are *N*-glycosylated enzymes and glycosylation contributes to proper folding and trafficking of the enzyme.[10, 11]

There are only few reports addressing successful production of recombinant human sialyltransferases in bacteria, mostly due to low yields of active, properly folded enzyme in this system. *E*. *coli* is the most popular organism for production of recombinant proteins due to the well-known advantages it offers over eukaryotic expression systems, i.e. fast growth rates, high final density cultures and low growth media costs.[12] However, eukaryotic proteins often require co- and post-translational modifications, which restricts their expression to the use of expensive systems such as yeast, Chinese Hamster Ovary (CHO) or insect cells. While glycosylation still remains a challenge for expression of native eukaryotic proteins in *E*. *coli*, some strategies have been developed to improve correct pairing of cysteines in recombinant proteins produced in this system. Such strategies include expression of recombinant proteins in the bacterial periplasm and the use of engineered strains with expression of redox-active enzymes to enable production of native disulfide bonds in the cytoplasm.[13] The use of engineered strains with the ability to handle correct oxidative protein folding in larger quantities in the cytoplasm i.e. Origami and SHuffle became popular in recent years. Pre- and co-expression of the chaperon/foldases yeast sulfhydryl oxidase (Erv1p) and protein disulfide isomerase (PDI) have also proven successful to enhance the yields of multi-disulfide bonded proteins in the cytoplasm of engineered and non-engineered *E*. *coli* strains.[14, 15]

In this work, we analyzed the contribution of solubility enhancer partners and the redox environment to the expression of functional disulfide bond containing human sialyltransferases ST3Gal1 and ST6Gal1 in *E*. *coli*. Activity of these enzymes is increased in different types of cancer,[2] and ST6Gal1 participates in the synthesis of core structure oligosaccharides of human milk oligosaccharides. Therefore, it is of utmost importance to generate enough sufficient amounts of fully functional STs to be applied in biochemical studies and synthetic processes. Here we showed that human ST3Gal1 and ST6Gal1 can be expressed in good yields in an economically viable bacterial system such as *E*. *coli*. Kinetic parameters both enzymes were obtained and they were successfully applied in the synthesis of sialosides from β-d-galactoside substrates.

## Results and Discussion

### Gene design and cloning of human sialyltransferases

STs from vertebrates have a type II transmembrane architecture characterized by a short *N*-terminal cytoplasmic tail followed by a 16–20 amino acids transmembrane domain, a flexible stem region and a *C*-terminal catalytic domain that orientates towards the luminal side.[8] Soluble *N*-terminal deletion variants of human ST6Gal1 and porcine ST3Gal1 lacking the transmembrane and stem regions were shown to be fully active.[11, 16, 17] Based on these results, codon optimized genes of hST3Gal1 and hST6Gal1 lacking the *N*-terminal cytoplasmic tail, the transmembrane domain and part of the stem region coding sequences were synthesized. Deletion variants of hST3Gal1 used in this work start from residues Thr35, Lys40 and Glu45 (Δ34, Δ39 and Δ44 variants). hST6Gal1 construct starts from residue Leu48 (Δ47 variant).

**Fig 1:**
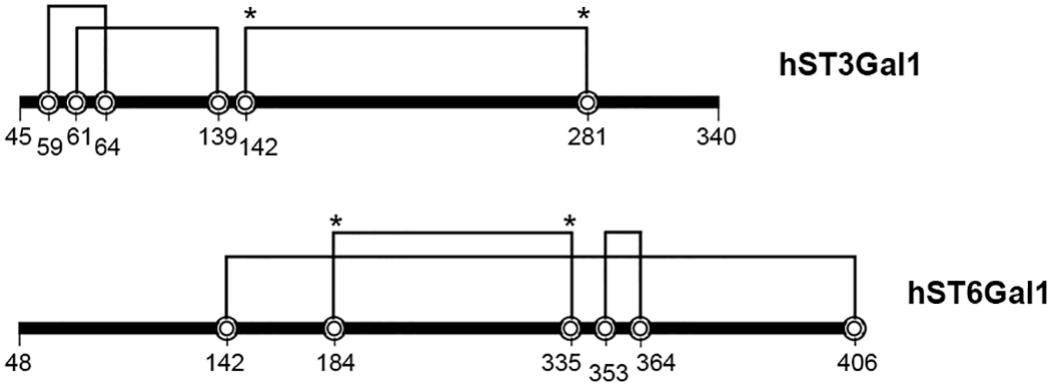
Scheme of disulfide bonds location on *N*-terminal truncated human ST3Gal1 and ST6Gal1. The bond formed between Cys142 and Cys281 in hST3Gal1 and Cys184 and Cys335 in hST6Gal1 (indicated by asterisks) is conserved in all STs from GT29.

Mammalian STs are *N*-glycosylated proteins containing both sequential and non-sequential disulfide bonds (Fig 1).[16, 18] The bond formed between Cys142 and Cys281 in hST3Gal1 and Cys184 and Cys335 in hST6Gal1 stabilizes the scaffold that shapes the CMP-Neu5Ac binding site and is conserved in all STs from GT29.[19] This bond is critical for catalysis, folding and transport, while other disulfide bridges are unique for each ST subfamily and their importance in STs activity varies.[19, 10] Glycosylation, on the other hand, seems not to be essential for activity of mammalian STS. It is, however, crucial for folding and stability.[11, 10] Due to such post-translational modifications, mammalian sialyltransferases have often been expressed in eukaryotic cells[11, 18, 10] and there are only few examples of successful expression of functional STs in bacteria.[16, 5] Previous attempts to obtain hST3Gal1 and hST6Gal1 in *E*. *coli* showed that even *N*-terminal deletion variants are poorly soluble and accumulate as inclusion bodies, yielding marginal activities or non-functional enzymes.[20, 21] To analyze the effect of both, solubility enhancer partners and redox environment on the expression of functional STs, we chose hST3Gal1 as a model. *N*-terminal variants Δ34, Δ39 and Δ44 were cloned into pETM-50 and pETM-80 for periplasmic expression in BL21. pETM-50 and pETM-80 include disulfide oxidoreductases (DsbA) or disulfide bond isomerase (DsbC) respectively as *N*-terminal tags.

hST3Gal1-Δ44 gene was cloned into pET28b^+^, pMAL-c5x and pLgals1-Tev[22] vectors encoding different *N*-terminal tags and genes were expressed in BL21, Origami2 DE3 and SHuffle *E*. *coli* strains.

Genes cloned in pET28b^+^ encode an *N*-terminal 6xHis-tagged protein. Genes cloned in pMAL-c5x have an *N*-terminal MBP- and a *C*-terminal 6xHis-tag. Genes cloned in pLgals1-Tev encode an *N*-terminal galectin-1-tagged sialyltransferase.

### Influence of redox environment and fusion partner in the expression of active hST3Gal1

A first strategy to obtain soluble and catalytically active hST3Gal1 involved the periplasmic expression of the three *N*-terminal variants Δ34, Δ39 and Δ44 fused either with DsbA or DsbC in the vectors pETM-50 and pETM-80 respectively. Medium additives such as sucrose, reduced glutathione, ethanol, arginine and sorbitol were included during STs expression in *E*. *coli* BL21 to promote solubility of the fusion proteins. These constructs resulted in insoluble protein and activity was not detected by High Performance Anion Exchange Chromatography with Pulsed Amperometric Detection (HPAEC-PAD) after incubation of soluble fractions over 2 h with 0.7 mm donor CMP-Neu5Ac and 0.4 mm acceptor benzyl 2-acetamido-2-deoxy-3-*O*-(β-d-galactopyranosyl)-α-d-galactopyranoside (Gal-β-1,3-GalNAc-α-O-Bn). Thus, cytoplasmic expression was not further pursued and experiments were performed with hST3Gal1-Δ44 hereafter (referred to as hST3Gal1 or MBP-hST3Gal1 for the MBP fused construct).

**Fig 2:**
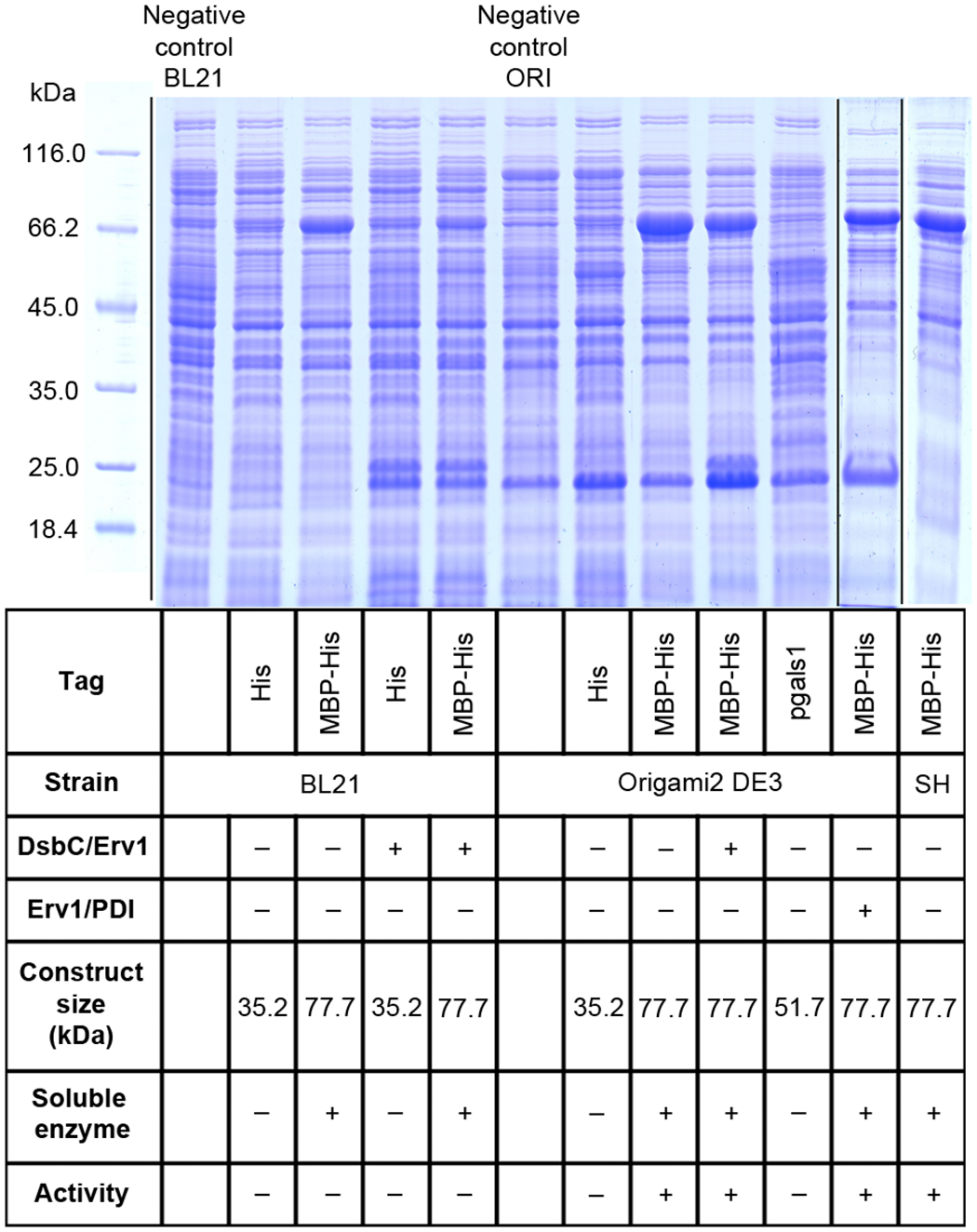
Production of soluble hST3Gal1 in the cytoplasm of *E*. *coli*. SDS-PAGE analysis of the expression and activity of His-, MBP/His- and galectin-1-tagged hST3Gal1 variants in different systems. SDS-PAGE image is composed of 4 gels, which is indicated by vertical black lines. SH: *E*. *coli* SHuffle.

Expression of a truncated *N*-terminal form of hST3Gal1 lacking 52 amino acids (hST3Gal1-Δ52) was previously reported in *E*. *coli* BL21(DE3)pLysS and *Pichia pastoris*. It was recovered as inclusion bodies in *E*. *coli* and the enzyme was inactive regardless the expression system.[20] We obtained similar results with the *N*-terminal His-tagged Δ44 variant expressed in the cytoplasm of *E*. *coli* BL21 (DE3), SHuffle and Origami2 strains (Fig 2). The recombinant enzyme was only observed as inclusion bodies and sialyltransferase activity was not detected in soluble fractions.

Fusion tags such as thioredoxin, small ubiquitin-like modifier proteins (SUMO), glutathione S-transferase (GST), disulfide oxidoreductase A (DsbA), galectin-1 and maltose-binding protein (MBP) have been widely used to improve solubility of recombinant proteins. Porcine ST3Gal1, which shares 85% sequence identity to its human homologue, was successfully expressed in the cytoplasm of *E*.*coli* Origami when fused with MBP. We fused human ST3Gal1 either to MBP or galectin-1 with only the former resulting in considerable amounts of soluble enzyme in the analyzed *E*. *coli* strains (Fig 2). Only MBP-fused enzymes expressed in *E*. *coli* strains with an oxidative cytoplasm, i.e. SHuffle and Origami showed activity.

Active human glycosyltransferases GalNAcT2 and ST6GalNAcI were recently expressed in engineered *E*. *coli* strains, containing either an oxidative cytoplasm or co-expressing the molecular chaperones/co-chaperones DnaK/DnaJ, trigger factor, GroEL/GroES and Skp.[5, 23] We analyzed the effect of chaperon/foldases co-expression on the activity of His- and MBP/His-tagged constructs in BL21 and Origami. Cells were co-transformed with the plasmid encoding hST3Gal1 (pMAL-5x) and a pMJS plasmid encoding either the pair sulfhydryl oxidase/protein disulfide isomerase (Erv1/PDI, plasmid pMJS9) or sulfhydryl oxidase/disulfide isomerase C (Erv1p/DsbC, plasmid pMJS10).[15] pMAL-5x and pMJS vectors belong to different incompatibility groups, which means they can be propagated in the same cell without competing for the replication machinery. pMAL-5x has the pMB1 origin of replication from pBR322, while pMJS vectors possess the p15A origin. It was previously shown disruption of reductive pathways in the cytoplasm of *E*. *coli* is not a strict requirement for the production of complex disulfide bonded eukaryotic protein when co-expressed with Erv1p/DsbC.[15] Contrary to these observations, we found a positive influence of chaperon/foldases only in Origami, but not in BL21 (Fig 2). This effect is discussed in later sections.

Not surprisingly, our results clearly show that both an oxidative environment and an appropriate solubility enhancer partner are needed to obtain functional hST3Gal1. Mutagenesis of invariant cysteine residues in ST6Gal1 and ST8Sia demonstrated that the disulfide bond formed between Cys142 and Cys281 (numbering in full length hST3Gal1) connects the conserved L (large) and S (small) sialyl motifs, which are involved in substrate binding. This bond is essential for maintaining an active conformation of the enzyme.[24, 10, 9] Cysteines from ST3Gl1have not been mutated; however, the bond between Cys142 and Cys281is expected to fulfill the same function in all GT29 STs, and analysis of the three-dimensional structure of pST3Gal1 shows correct pairing of structurally close Cys59, Cys61, Cys64 and Cys139 which are located near the *N*-terminus is most likely critical for maintaining the native fold.

Our results show that a favorable oxidative environment is not the sole requirement to achieve a functional folding. According to previous expression studies of sialyltransferases in bacterial and eukaryotic cells, glycosylation seems to play a major role in acquiring a native fold. However, this role varies among enzymes from the ST3Gal subfamily. For example, fully deglycosylated pST3Gal1 retains folding and function.[16] hST3Gal1 has five potential *N*-glycosylation sites with four of them (Asn79, Asn114, Asn201 and Asn323) in the catalytic domain. A fully deglycosylated hST3Gal1and a mutant lacking the first three glycosylation sites showed reduced activity and poor expression in insect cells.[10] Two forms of the hST3Gal1-Δ52 variant, i.e. a fully deglycosylated and a glycosylated (high mannose type) form were found inactive when expressed in *P*. *pastoris*.[20] In this work, fusion of a deglycosylated hST3Gal variant with MBP, but not with galectin-1, DsbA or DsbC proved effective for solubility, which in combination with a proper environment for disulfide bonds formation resulted in significant amounts of active enzyme. To our knowledge, this is the first report of the expression of a functional hST3Gal1 in bacteria.

### Expression of MBP-hST3Gal1 in SHuffle and Origami strains

**Fig 3:**
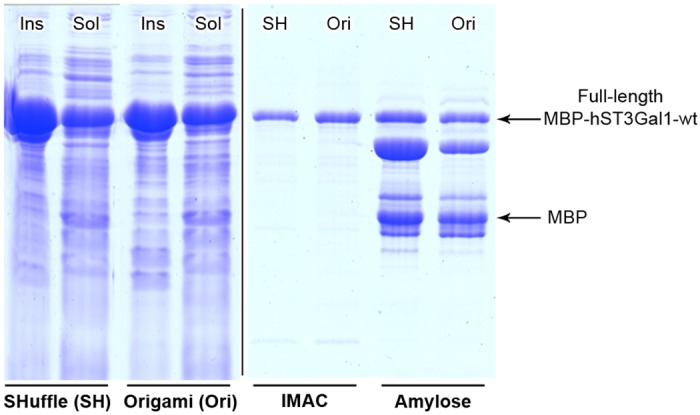
SDS-PAGE of the expression of MBP-hST3Gal1 in SHuffle (SH) and Origami (Ori). Expression of MBP-hST3Gal1 and its purification from each system via IMAC and amylose resin are shown. Insoluble (Ins) and soluble (sol) fractions are indicated.

A strong expression of the fusion enzyme MBP-ST3Gal1was observed in SHuffle and Origami, with a major fraction of the enzyme found as inclusion bodies regardless the expression conditions, i.e. inducer concentration and growth temperature after induction (Fig 3) Enzymes analyzed in this work were expressed with 100 μm IPTG at 17°C and 200 rpm shaking. A continuous spectrophotometric assay was performed to determine sialyltransferases activity using CMP-Neu5Ac as donor and Gal-β-1,3-GalNAc-α-*O*-Bn as acceptor. About 18 and 10 units of enzymatic activity were measured in cleared lysates per liter of culture of SHuffle and Origami respectively. Higher activity was detected in SHuffle due to the faster growth of this strain under the described conditions, which resulted in more biomass after 22 h. SHuffle and Origami reached a final OD_600_ of 4.2 and 2.2 respectively.

Immobilized metal ion affinity chromatography (IMAC) and one-step purification using affinity of MBP for amylose were performed to recover MBP-hST3Gal1from cleared lysates. MBP-hST3Gal1 was readily purified by IMAC but not via MBP’s affinity most likely due to instability of the fusion protein, as some bands between the size of MBP (42.5 kDa) and the size expected for the full-length fusion protein (77.7 kDa) were co-purified (Fig 3). SDS-PAGE analysis of initial cleared lysates, flow-through and elution fractions revealed that MBP-hST3Gal1 did not bind quantitatively either to IMAC or amylose resins (S1 Fig). Additives such as glycerol, NaCl, glycine, urea, tween-20, triton X-100 and IGEPAL CA-630 were added to resuspended cells before lysis, aiming to disrupt possible intermolecular interactions and thus enhance solubility and stability of MBP-hST3Gal1. Only glycerol had some effect on protein recovery by IMAC (S2 Fig). With glycerol added before cell lysis, 3 and 4 mg of IMAC-purified MBP-hST3Gal1 were obtained per liter culture of Origami and SHuffle respectively. Only half of the sialyltransferase activity measured in SHuffle cleared lysates could be recovered by IMAC. Furthermore, low reproducibility in the yields of active enzyme was observed from batch to batch when using this system; therefore expression studies were continued exclusively with Origami.

**Fig 4:**
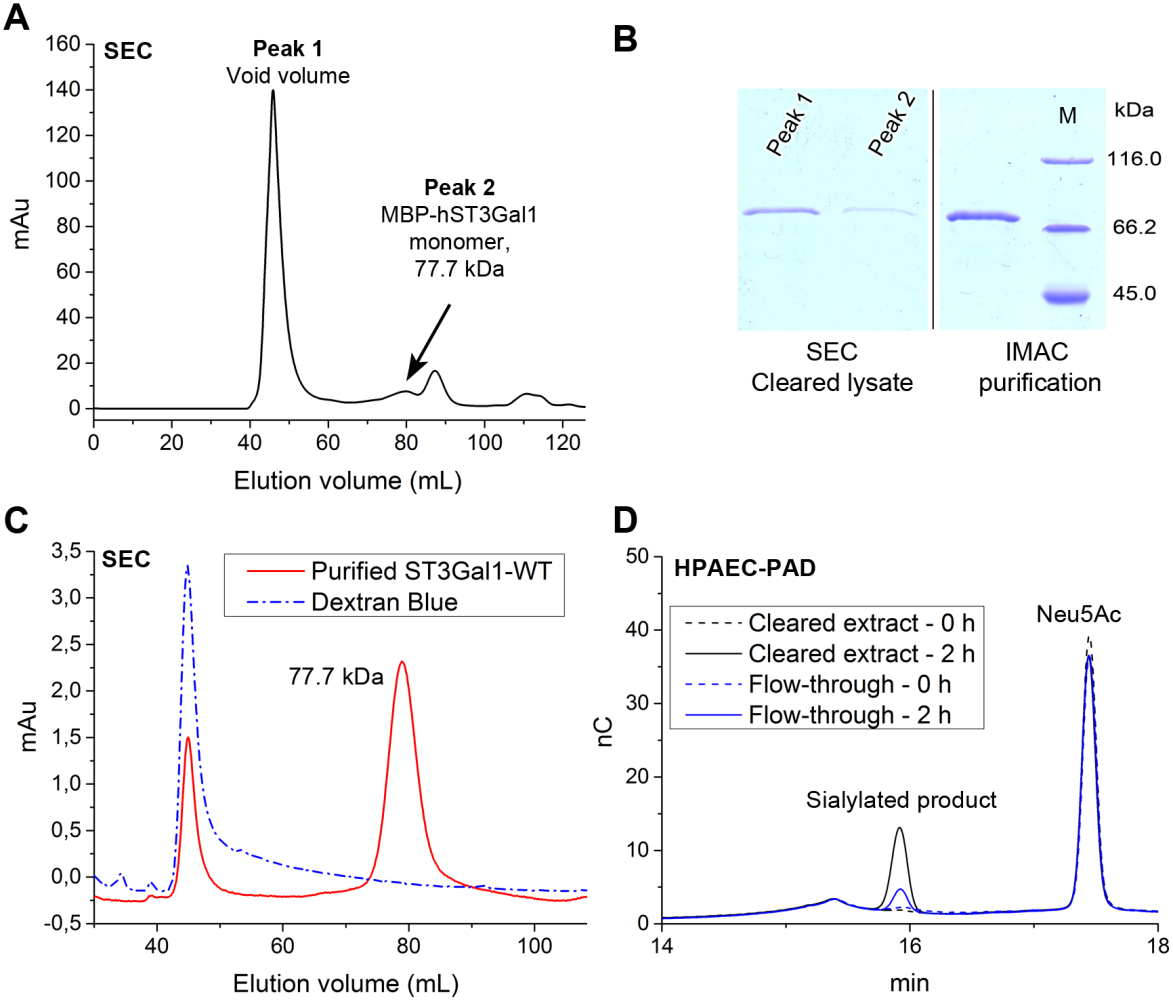
Analysis of soluble MBP-hST3Gal1 expressed in Origami. A) SEC profile of cleared cell lysate containing overexpressed MBP-hST3Gal1 (black). B) SDS-PAGE of the IMAC purified fusion protein and two SEC elution fractions (peaks 1 and 2) from the cleared cell lysate. C) Chromatogram of dextran blue, which elutes in the void volume (blue), and IMAC purified MBP-hST3Gal1 (red). Monomeric MBP-hST3Gal1 elutes at around 80 mL volume. D) HPAEC-PAD chromatogram showing activity of the cleared cell lysate containing MBP-hST3Gal1 before (black) and after (flow-through fraction, blue) incubation with IMAC. Activity was determined in the synthesis of 3’-sialyl-Gal-β-1,3-GalNAc-α-*O*-Bn after 0 and 2 h incubation (dashed and continuous lines respectively) with CMP-Neu5Ac and Gal-β-1,3-GalNAc-α-*O*-Bn. M: protein molecular weight standard. Original SDS-PAGE image was cropped for clarity (indicated by a vertical black line), as displayed fractions were in non-adjacent lanes. Only the upper part of the gel demonstrating the presence of sialyltransferase is shown.

MBP fusions often result in soluble heterogeneous multimeric aggregates and frequently only a small fraction of the fusion protein is properly folded and active.[25] The size-exclusion chromatography (SEC) profile of cleared cell lysates containing MBP-hST3Gal1 shows a heterogeneous distribution of the fusion enzyme, with most of MBP-hST3Gal1 eluting in the void volume (Fig 4A and 4C). Although IMAC purified MBP-hST3Gal1 is soluble and active, the enzyme also elutes as two peaks, with a major fraction eluting in the volume corresponding to the monomer (Fig 4B). Activity assays performed by HPAEC-PAD of cleared lysates before and after incubation with IMAC resin showed most of the sialyltransferase activity was recovered by purification (Fig 4D). As a significant proportion of soluble MBP-hST3Gal1 is inactive, it could be concluded that around 90% of the soluble recombinant MBP-hST3Gal1 produced in Origami is misfolded, while the remaining enzyme shows sialyltransferase activity and is found as a heterogeneous (monomeric and oligomeric) distribution.

Specific activity of purified MBP-hST3Gal1 was determined to be 2.3 μmol mg^-1^ min^-1^ (± 0.15) and was consistent in different purification batches, indicating that the same fraction of active enzyme was recovered by purification (S3 Fig).

### Pre- and co-expression of MBP-hST3Gal1 and MBP-hST6Gal1 with chaperon/foldases in Origami

Pre-expression of chaperon/foldase systems was previously reported to be beneficial for the production of a fragment of soluble folded plasminogen activator (vtPA), which contains nine disulfide bonds.[15] Pre-expression would result in an early production of folding factors, which would be available once expression of the disulfide bonded protein is started. The same strategy was applied in this work to increase the population of folded MBP-hST3Gal1. Expression of chaperon/foldases was induced with 0.5% (l)-arabinose at an OD_600_ of around 0.4, followed by induction of MBP-hST3Gal1 with 100 μM IPTG at an OD_600_ ≈ 0.6. Pre-expression of folding factors was carried out at 30°C for 1 h and expression of STs at 17°C for 22 h.

**Fig 5:**
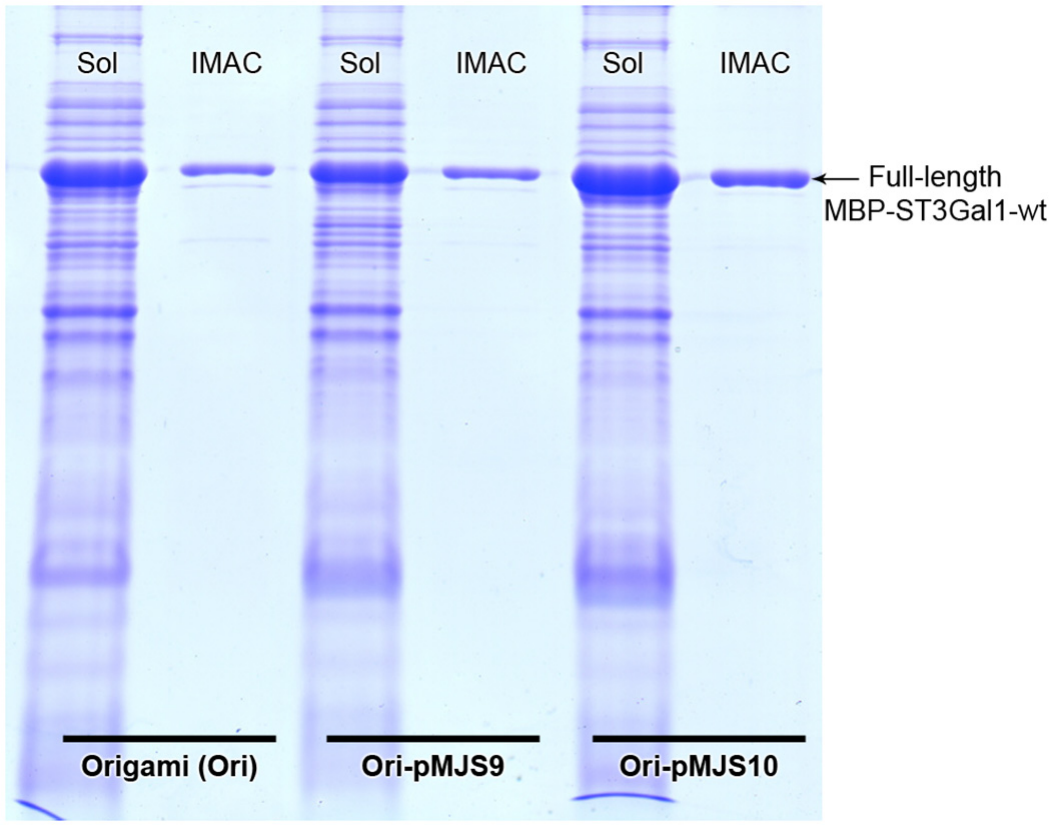
Expression of MBP-hST3Gal1 in Origami with and without folding factors. SDS-PAGE of the expression of MBP-hST3Gal1 in Origami (Ori) and co-expression of the fusion enzyme with Erv1/PD (Ori-pMJS9) and Erv1p/DsbC (Ori-pMJS10). IMAC purified enzyme from each system is also shown.

Expression of MBP-hST3Gal1 in Origami with and without co-expression of chaperon/foldases was compared. Protein concentration was determined in cleared lysates by the Bradford method and same amount of protein was loaded onto IMAC purification columns. Cleared lysates and flow-through and pooled elution fractions were analyzed by SDS-PAGE (Fig 5). About 40% more activity was observed in cleared soluble lysates co-expressing Erv1p/DsbC and 20% more protein was recovered by IMAC purification when compared to the yields of MBP-hST3Gal1 in absence of folding factors. In average 2.5 and 3 mg of IMAC purified MBP-hST3Gal1 were recovered per liter of culture when co-expressed with Erv1/PDI and Erv1p/DsbC, respectively. Reported yields are the result of at least 5 independent expression experiments. Although a larger population of active sialyltransferase is detected in the system co-expressing Erv1p/DsbC when compared on the bases of mg of total soluble protein in cleared lysates, the yield of IMAC purified protein per liter of culture is the same than that obtained from Origami without co-expression of folding factors. Co-expression of chaperon/foldases causes a metabolic burden on *E*. *coli*, which results in increased duplication times, and hence in less biomass per liter of culture (final OD_600_ ≈ 1.7) after 22 h expression, compared to biomass yields in Origami producing only MBP-hST3Gal1 (final OD_600_ ≈ 2.2).

**Fig 6:**
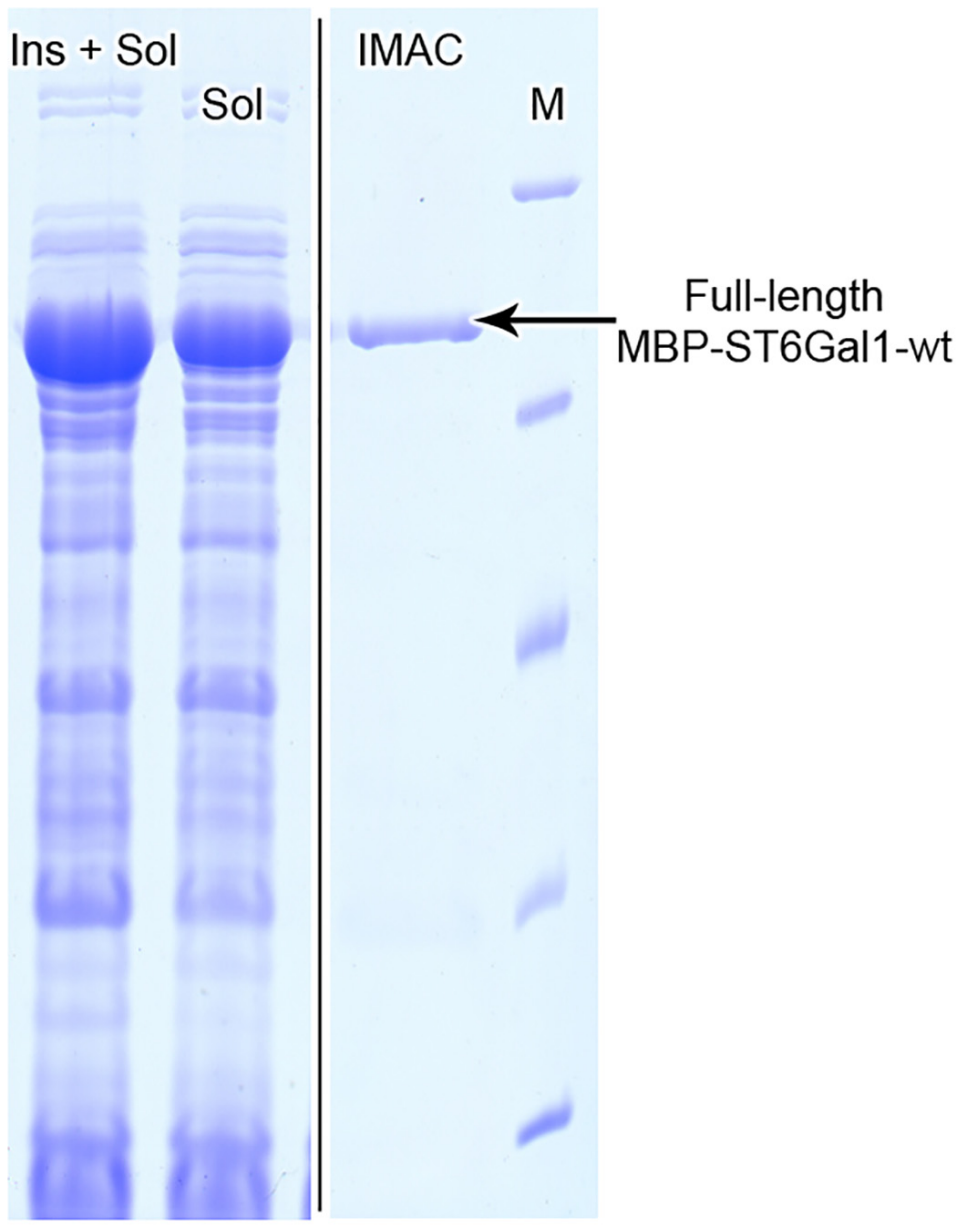
SDS-PAGE of the co-expression of MBP-hST6Gal1 with Erv1p/DsbC (pMJS10) in Origami. Lysate after cell disruption (Ins + sol), cleared lysate (sol) and IMAC purified enzyme are shown. M: protein molecular weight standard. Original SDS-PAGE image was cropped for clarity, as indicated by a vertical black line.

Expression of the human sialyltransferase MBP-hST6Gal1 in Origami co-expressing Erv1p/DsbC (Fig 6) showed similar results to those obtained for hST3Gal1. 2 mg of MBP-hST6Gal1 were obtained by IMAC purification and the specific activity of the fusion protein was determined to be 2.0 μmol mg^-1^ min^-1^ when assayed with 0.7 mm CMP-Neu5Ac and 10 mm *N*-acetyllactosamine. Expression of an *N*-terminal truncated hST6Gal1 fused with MBP in the cytoplasm of non-engineered *E*. *coli* was previously reported. Authors reported a yield of 266 μg of purified fusion protein per liter of culture.[21] The yield of purified and active MBP-hST6Gal1 reported in this work (2 mg L^-1^culture) is 8-times higher than that reported for a non-engineered *E*. *coli* strain, most likely due to the improvement of native disulfide bonds formation resulting from both, the oxidative cytoplasm of Origami and co-expression of folding factors.

In summary, production of soluble although inactive MBP-hST3Gal1 in BL21 showed the necessity of disulfide bonds formation to obtain active enzyme in *E*. *coli*. Expression in Origami resulted in good yield of active STs, which was increased in presence of folding factors. Although the effect of Erv1p/DsbC was smaller than that observed for proteins previously reported,[15] this result indicates that mispaired cysteines resulting in wrong disulfide bridges may account for a fraction of misfolded protein in absence of Erv1p/DsbC.

### A supercharging-like strategy for folding and solubility

**Fig 7:**
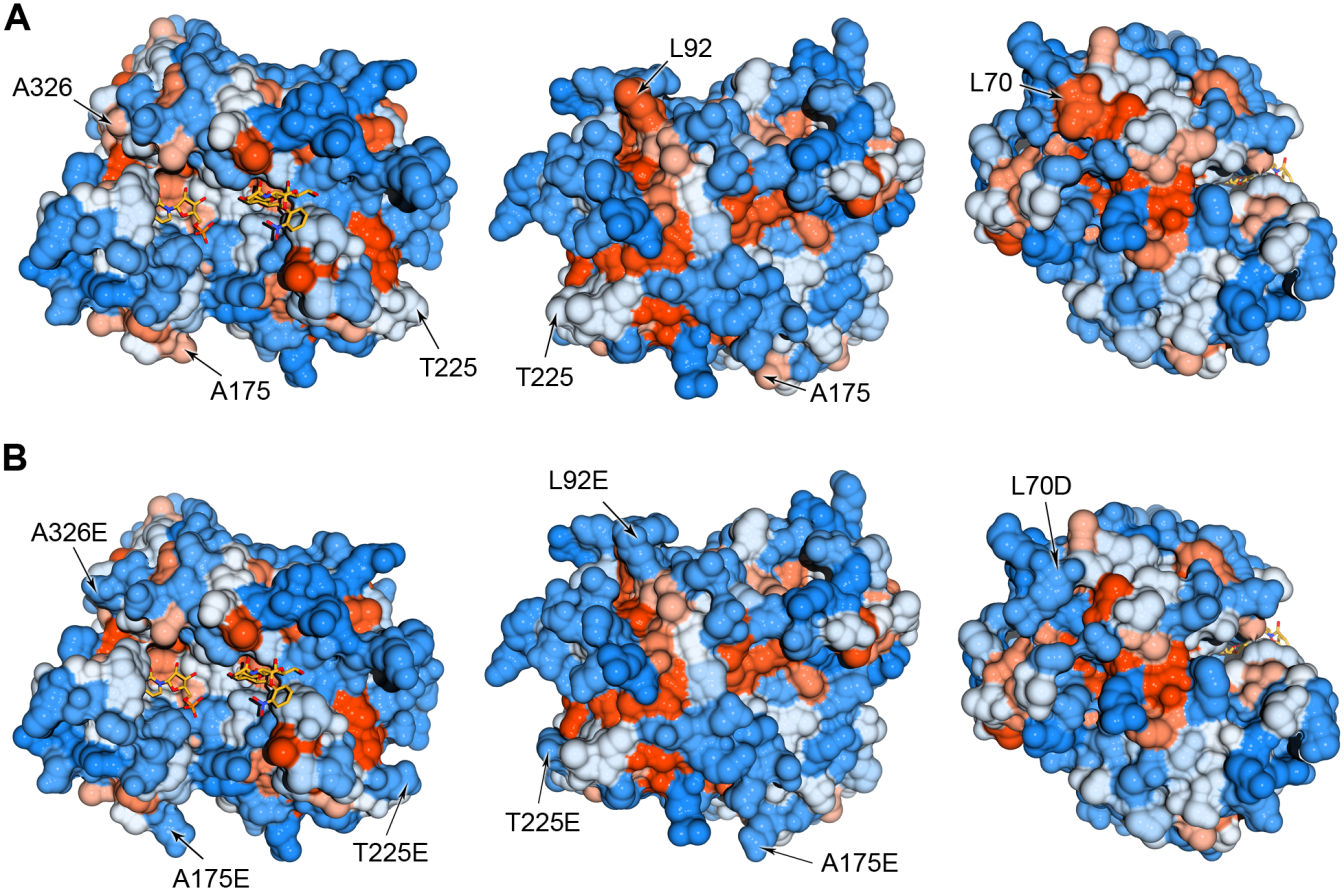
Models of wild type hST3Gal1 (A) and variant hST3Gal1-5x (B). Position of mutations L70D, L92E, A175E, T225E and A326E are indicated by arrows. The scale for hydrophobicity shows amino acids with colors ranging from blue for the most hydrophilic to orange red for the most hydrophobic. Figures and *in silico* mutations on the model were created using UCSF Chimera[29].

Despite optimization of the redox environment and protein solubility, a large proportion of recombinant hST3Gal1 does not acquire the native fold. Thus, a different strategy was sought to drive hST3Gal1folding to its active native state. Supercharging, defined as the increase in the net charge of a protein by introducing changes to its exposed residues via mutagenesis, is a strategy that has been used to increase solubility of various proteins expressed in *E*. *coli* and to assist reversible unfolding.[26, 27] The rationale behind protein supercharging is the prevention of ordered and disordered aggregation by disruption of non-specific interactions and by favoring charge repulsion between molecules.[28] In order to avoid destabilization of the folding state and to retain the native structure, solvent-exposed flexible polar residues and surface hydrophobic residues are often “hotspots” for mutagenesis. Aiming to prevent partially unfolded states, we followed the second strategy by removing surface hydrophobic residues.[28] A three dimensional model of hST3Gal1 was generated by the SWISS-MODEL server (http://swissmodel.expasy.org/) using its porcine homologue [PDB: 2WNB] as a template and exposed hydrophobic residues were identified. Amino acids located at short hydrophobic regions were chosen for mutagenesis and a variant of hST3Gal1 with the mutations L70D, L92E, A175E, T225E and A326E was constructed (Fig 7). All mutations are located far from the active site. The number of negatively charged residues was increased from 33 in the native sequence to 38 in the variant hST3Gal1-5x. Since the protein net charge at pH 7.0 was decreased from 10.8 to 5.8 (Protein calculator, Innovagen), we call the substitution of exposed hydrophobic amino acids in our variant a supercharging-like approach.

**Fig 8:**
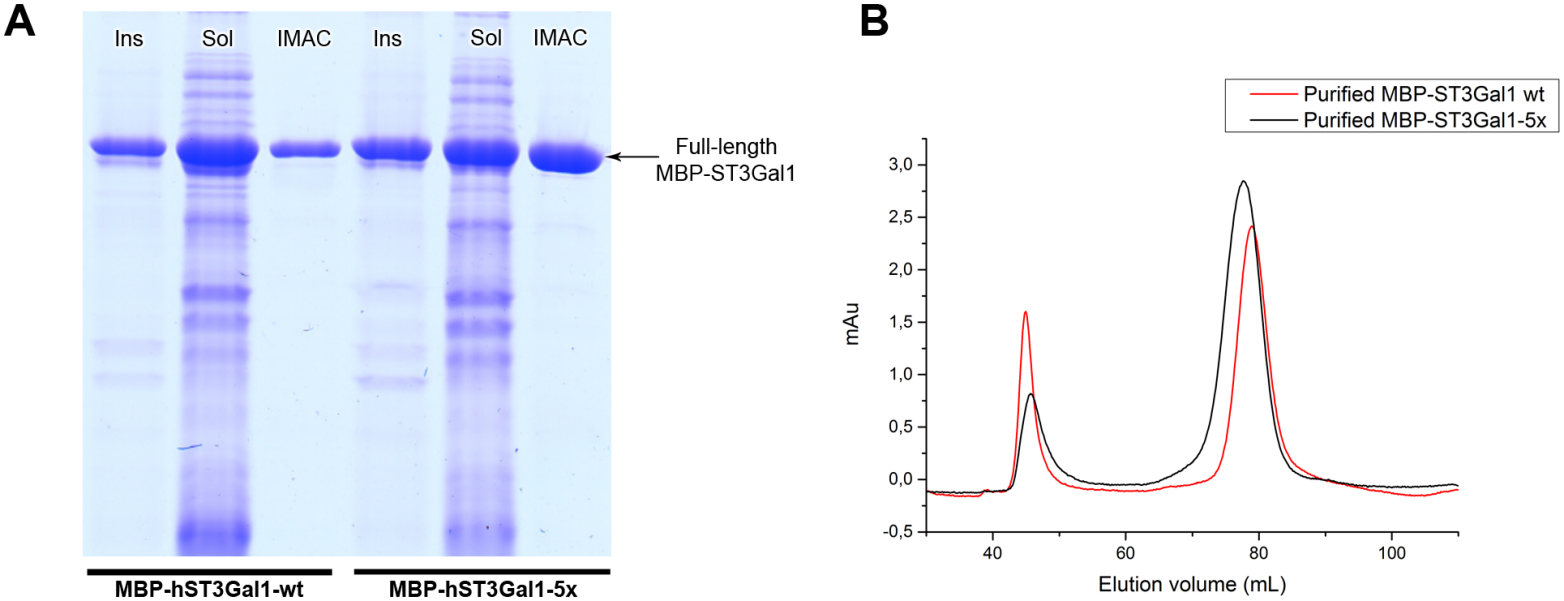
Comparison of expression and purification of MBP-hST3Gal1-wt and MBP-hST3Gal1-5x. The fusion proteins were produced in Origami with co-expression of Erv1p/DsbC. Insoluble (Ins), soluble (sol) and IMAC purified fractions are indicated. B) SEC profile of IMAC purified MBP-hST3Gal1-wt and MBP-hST3Gal1-5x.

MBP-hST3Gal1-wt and the quintuple variant (MBP-hST3Gal1-5x) were expressed under the same conditions in Origami co-expressing Erv1p/DsbC. Around 7 mg of the quintuple mutant were recovered by IMAC purification per liter of culture; this is 2.3-times more protein than the wild type enzyme (3 mg L^-1^ culture) (Fig 8A). As expected, less MBP-hST3Gal1-5x was observed in the flow-through fraction when analyzed by SDS-PAGE (S1 Fig).

Specific activity of the variant was similar to that of the wild type enzyme (2.4 and 2.3 μmol mg^-1^ min^-1^ respectively); indicating the activity of hST3Gal1 was not affected by the mutations.

Notwithstanding the higher activity observed in cleared extracts containing the quintuple variant, SEC profiles of cleared lysates and IMAC purified protein are similar to those of the wild type enzyme. The fraction of oligomeric purified hST3Gal1-5x, however, is smaller than that of the wild type enzyme, with the population of the monomeric enzyme increased (Fig 8B). A plausible explanation for the higher activity observed in cleared lysates containing the variant and the improved recovery of purified enzyme is that mutations could have influence both, folding and the number of molecules involved in protein self-association, improving IMAC binding due to the modified 6xHis tag availability.

In order to analyze the contribution of the mutations to the native fold, expression of MBP-free (referred to as His-hST3Gal1-5x) and MBP-fused constructs of the quintuple mutant was analyze in BL21. Interestingly, marginal activity (close to the HPAEC-PAD detection limit) was observed for both constructs when expressed in BL21 (S4 Fig). Similar wild type constructs are inactive in BL21, which indicates that mutations may have indeed improved protein folding. Initially, low activity observed for His-hST3Gal1-5x in BL21 was attributed to the low concentration of soluble enzyme in cleared extracts, however, the highly soluble MBP-hST3Gal1-5x construct showed similar low activity (S4 Fig). Since the variant MBP-hST3Gal1-5x is highly active when expressed in Origami, these results suggest *E*. *coli* BL21 is able to handle correct oxidation of only a minor proportion of hST3Gal1-5x.

Low production of soluble His-hST3Gal1-5x was also observed in Origami (S4 Fig), which shows substitution of exposed hydrophobic residues and an optimal oxidative environment is not sufficient for protein folding in the absence of a solubility enhancer. Therefore, the effects of an optimized oxidative cytoplasm, solubility enhancer tag (MBP) and surface charge modification may be additive for the production of a larger proportion of correctly folded and active hST3Gal1.

Additional supercharging protocols may be explored in future to further improve the yields of these and other STs in *E*. *coli*.

### Analysis of the secondary structure of hST3Gal1 and hST6Gal1 by circular dichroism

IMAC purified MBP-STs were incubated with Factor Xa protease, which cleaves after the arginine residue in the cleavage site Ile-Glu-Gly-Arg to release MBP.

**Fig 9:**
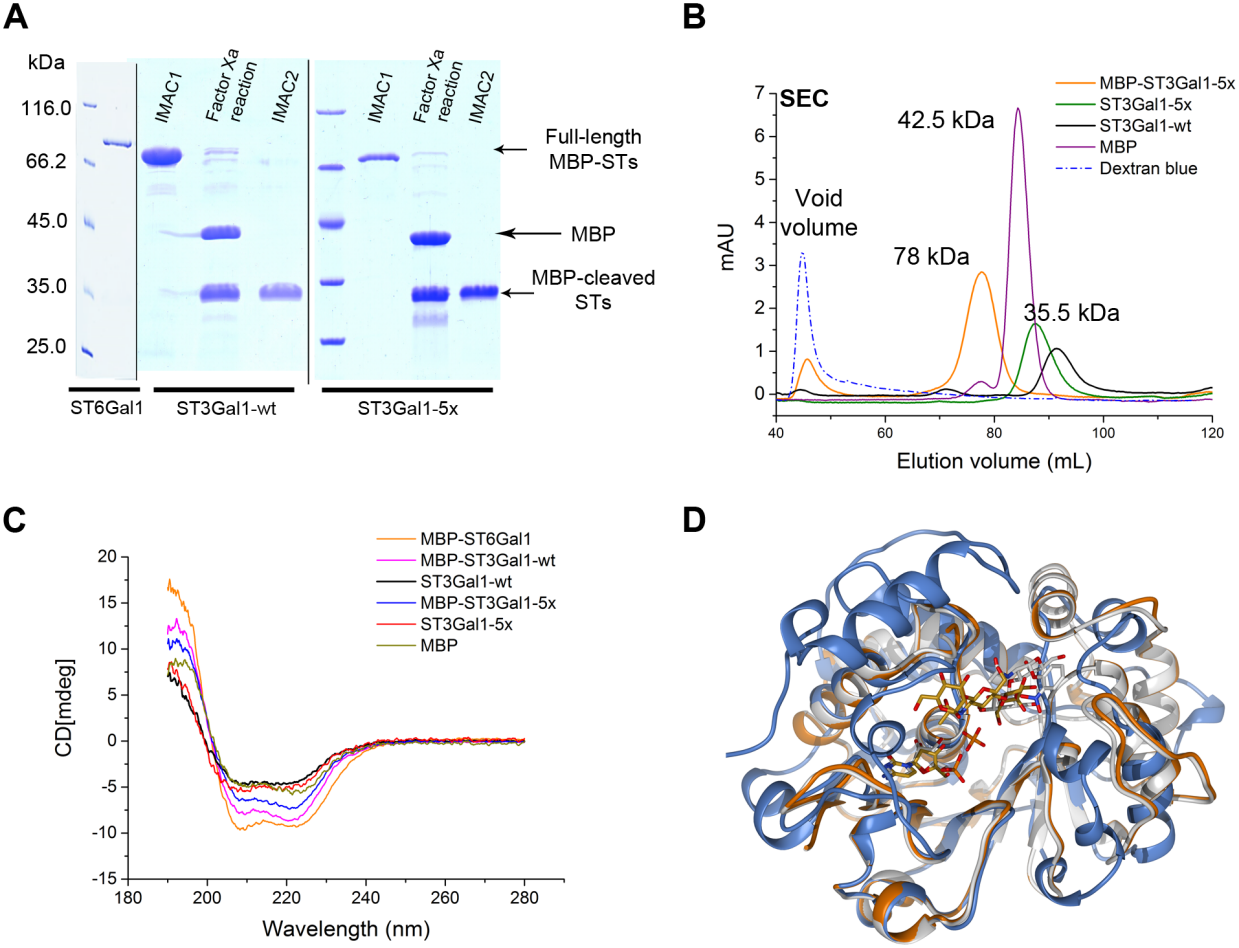
Structural analysis of MBP-fused and MBP-cleaved STs. A) SDS-PAGE of MBP-fused and MBP-cleaved STs. IMAC1: purification of full length fusion proteins; IMAC2: purification of MBP-cleaved STs after incubation with Factor Xa protease. B) SEC profile of IMAC purified MBP-hST3Gal1-5x and MBP-cleaved hST3Gal1-wild type and -5x variant. Dextran blue indicating the void volume and MBP are also shown. C) Circular dichroism spectra of MBP, MBP-STs and MBP-cleaved STs. D) Tertiary structure of hST6Gal1 (blue, PDB: 4js1)[18], pST3Gal1 (white, PDB: 2wnb)[16] and hST3Gal1 (model based on PDB 2wnb). SDS-PAGE image is composed of 3 gels, which is indicated by vertical black lines.

Cleaved proteins were re-buffered and STs were purified by IMAC via their *C*-terminal His-tag. Factor Xa and released MBP do not bind to the IMAC resin. hST3Gal1-wt and hST3Gal1-5x were found to be stable after cleavage and remained in solution upon separation from MBP (Fig 9A). Unlike their MBP-fused counterparts, cleaved hST3Gal1-wt and hST3Gal1-5x are only found as monomers (Fig 9B), which indicate both, STs and MBP domains may be involved in the aggregation process described above even for the fraction of folded and active fusion protein.

Insufficient amounts of cleaved ST6Gal1 were recovered by IMAC purification. Consequently, further characterization of this enzyme was performed with the fusion protein.

Secondary structure of fusion and MBP-cleaved STs was assessed by circular dichroism spectroscopy.

As shown in Fig 9C, sialyltransferases display the characteristic spectrum of a folded mostly helical protein: negative bands at 222 and 208 nm and a positive band at 193 nm.[30] According to their three dimensional structure, pST3Gal1 and hST6Gal1 have a mixed αβ fold, composed of 7 twisted β-strands flanked by 12 α-helices.[16, 18] β-strands occupy similar locations in ST3Gal1 and ST6Gal1 enzymes, and differences are observed in the helical and loop segments that constitute the rest of the structure of both enzymes, including the acceptor-binding site.[19, 18, 16] Tertiary structure of hST3Gal1-wt (model), pST3Gal1 [PDB: 2WNB] and hST6Gal1 [PDB: 4js1] are shown in Fig 9D.

Circular dichroism results indicate that the active fraction of STs expressed in *E*. *coli* and recovered by IMAC purification has an ordered secondary structure. The quintuple hST3Gal1 mutant shows a CD spectrum similar to that of the wild type enzyme, which confirms that modification of five exposed hydrophobic amino acids does not affect the structure of the variant.

### Kinetic studies

ST3Gal1 enzymes from family GT29 transfer sialic acids via α2,3 linkages onto a terminal galactose found in type 3 oligosaccharides Galβ1-3GalNAc-R or in the oligosaccharide Galβ1-3/4GlcNAc-R, attached to glycoproteins and glycolipids.[31] ST6Gal proteins form α2,6 linkages between sialic acids and the acceptor, transferring sialic acids to *N*-glycans bearing the outer type 2 disaccharide Galβ1,4GalNAc.[7]

Kinetic parameters for donor and acceptor substrates were obtained for hST3Gal1 and hS3Gal1-5x in reactions containing Gal-β-1,3-GalNAc-α-*O*-Bn as acceptor and CMP-Neu5Ac as donor (Table 1).

**Table 1:** Kinetic parameters of mammalian sialyltransferases.

Enzyme	Donor CMP-Neu5Ac			Acceptor Galb1,3GalNAc-a-OBn			
	K_M_ (μm)	K_cat_ (min^-1^)	Kcat/K_M_ (mm^-1^ min^-1^)	K_M_ (μm)	K_cat_ (min^-1^)	K_cat_/K_M_ (mm^-1^ min^-1^)	
MBP-hST3Gal1-wt	106 (± 15)	201 (± 7)	1896	26 (± 7)	189 (± 2)	7214	This work
hST3Gal1-wt	98(± 13)	153 (± 6)	1563	40 (± 7)	160 (± 6)	4009	This work
MBP-hST3Gal1-5x	132 (± 19)	199 (± 10)	1504	24 (± 2)	221 (± 5)	9212	This work
hST3Gal1-5x	116 (± 19)	185 (± 10)	1592	35 (± 7)	168 (± 7)	4811	This work
hST3Gal1Δ56	14	N.D		23	N.D		[17]
MBP-pST3Gal1	123 (± 11)	170 (± 5)	1382	70 (± 10)	172 (± 7)	2430	[32]
pST3Gal1	90	152	1620	70	170	2530	[16]
MBP-hST6Gal1	62 (± 6)	81 (± 2)	1318	N.D	N.D	N.D	This work
ratST6Gal1	92 (± 10)	36 (± 0.3)	387	1.8 (± 0.3)	40 (± 0.8)	22.4	[19]

k_cat_ and K_M_ parameters obtained for donor CMP-Neu5Ac are consistent with those previously reported for the porcine sialyltransferase using the same donor and acceptor substrates and similar conditions for activity detection (a multi-enzymatic assay coupled to NADH oxidation). While still in the same order of magnitude, K_M_ values for the acceptor are lower for human MBP-hST3Gal1 than those reported for porcine MBP-pST3Gal1 (26 and 70 μM respectively).[16, 32] Kinetic parameters derived from a radioactive assay were published previously for an *N*-terminal truncated hST3Gal1 (hST3Gal1Δ56) expressed in COS-7 cells (Table 1).[10] Although K_M_ values reported for MBP-cleaved hST3Gal1 (variant Δ44 in this work) and hST3Gal1Δ56 for the acceptor are similar (40 and 23 μm respectively), values for the donor greatly differ (98 and 14 μm respectively). A direct comparison could not be established between hST3Gal1 enzymes due to the different assays that were performed for activity detection, however these results together with the secondary structure analysis suggest the fraction of hST3Gal1 expressed in *E*. *coli* and recovered by IMAC is folded and entirely functional, which enables the enzyme to be used in biochemical and structural studies.

Kinetic behavior of MBP-hST3Gal1-wt and MBP-hST3Gal1-5x are similar, and values obtained from MBP-fused and MBP-cleaved forms are comparable as well (Table 1 and S5 Fig).

It was not possible to obtain K_M_ and k_cat_ values for hST6Gal1 using LacNAc as acceptor, since saturation was not observed under the assayed conditions. However, values obtained for the donor CMP-Neu5Ac are in the range of those reported for its homologue from rat. A comparison of the kinetic parameters of different STs is shown in Table 1.

### Sialylation of β-D-galactosides

**Fig 10:**
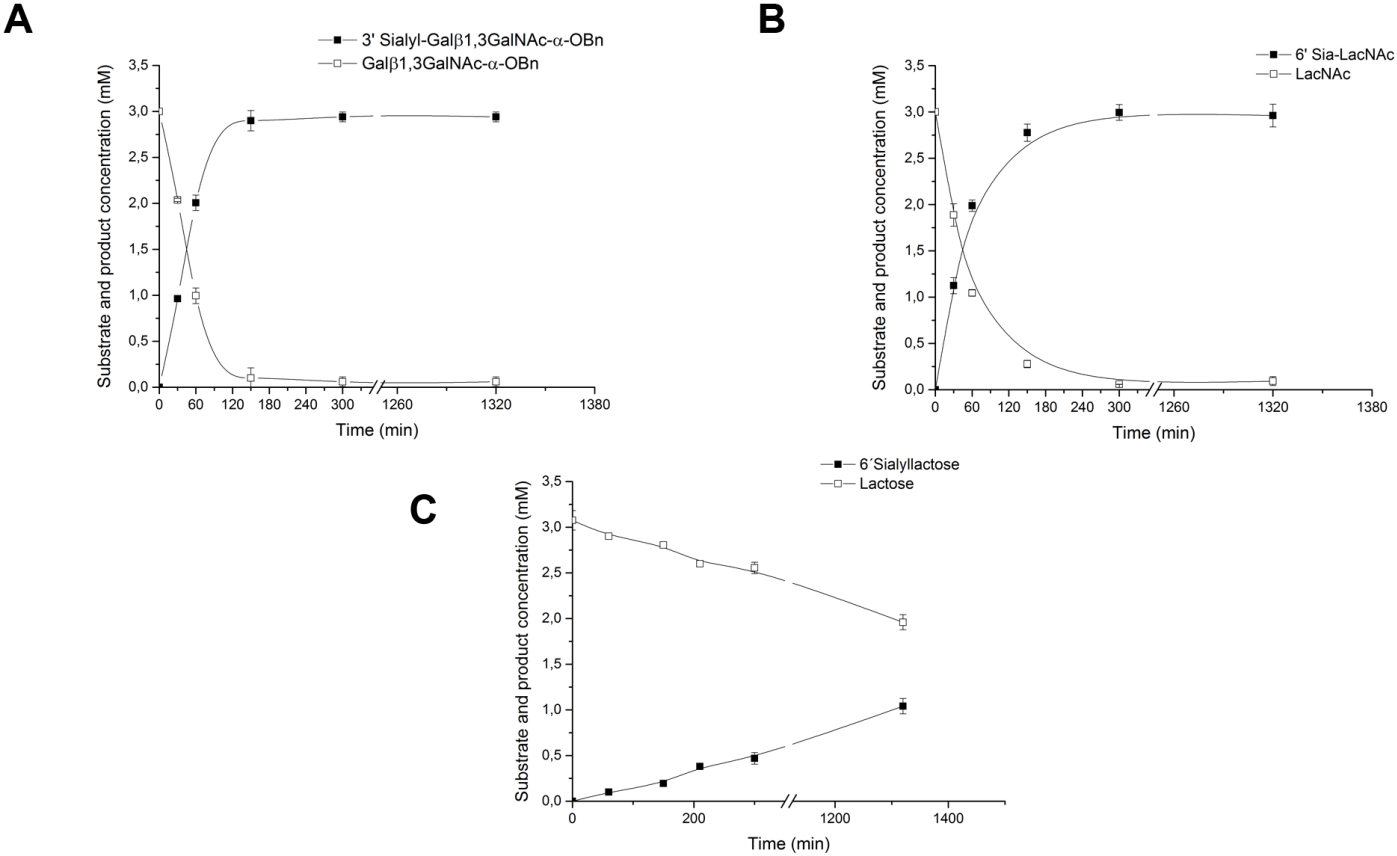
Synthesis of sialosides by MBP-ST3Gal1 and MBP-ST6Gal1. Reactions containing 4.50 mm CMP-Neu5Ac (donor), 3.0 mm acceptor (Gal-β-1,3-GalNAc-α-*O*-Bn, lactose or *N*-acetyllactosamine) and sialyltransferases at a final concentration of 1.0 or 2.0 μm where prepared in 20.0 mM MOPS, pH 7.5 in 40.0 μL final volumes. Reactions were incubated at 37°C and samples analyzed over the reaction time of 22 h. Progress in the synthesis of 3’-sialyl-Gal-β-1,3-GalNAc-α-*O*-Bn (A), 6’- sialyl-*N*-acetyllactosamine (B) and 6’- sialyllactosamine was monitored by HPAEC-PAC.

MBP-fused hST3Gal1 and hST6Gal1 were applied in the synthesis of sialosides from β-d-galactoside acceptors such as lactose, *N*-acetyllactosamine and Gal-β-1,3-GalNAc-α-*O*-Bn. Reactions were carried out with 4.5 mm CMP-Neu5Ac, 3 mm acceptor and 1 μm enzyme (unless otherwise specified). Reaction progression was followed over the time course and samples were analyzed by HPAEC-PAD. Conversion of substrates and yields of products were calculated using appropriate commercial standards. As previously mentioned, the mucin type disaccharide Gal-β-1,3-GalNAc found on glycolipids or *O*-glycosyl proteins is the best acceptor for ST3Gal1 enzymes,[32, 10] thus we used the commercially available substrate Gal-β-1,3-GalNAc-α-*O*-Bn to analyze the performance of MBP-hST3Gal1 in the synthesis of 3’-sialosides. MBP-hST3Gal1 was also applied in the synthesis of 3’-sialyllactose and 3’-sialyl-*N*-acetyllactosamine using 2 μm enzyme. As expected, hST3Gal1 was highly efficient with Gal-β-1,3-GalNAc-α-*O*-Bn as acceptor, obtaining quantitative yields of the sialylated product (Fig 10A). Yields of 16 and 25% were obtained in the synthesis of 3’-sialyllactose and 3’sialyl-*N*-acetyllactosamine respectively. Vertebrate ST3Gal1 enzymes show low K_M_ and k_cat_ values for lactose and *N*-acetyllactosamine, which, together with the lability of donor CMP-Sia, result in considerable donor hydrolysis over large incubation times and low product yields. Previous studies showed galactosides bound through β-1,3-linkages to glycoside moieties other than *N*-acetylgalactosamine and those bound through β-1,4-linkages are very poor acceptors for the porcine ST3Gal1.[33, 32] In fact, other enzymes from the ST3Gal subfamily such as ST3Gal4 catalyze the formation of a α-2,3-linkages between Neu5Ac and terminal galactose residues found on *N*-acetyllactosamine motifs in glycoproteins and glycolipids.[7] The supercharged variant of hST3Gal1 behaves as the wild type enzyme in the synthesis of sialosides.

MBP-hST6Gal1 was applied in the synthesis of 6’-sialyllactose and 6’sialyl-*N*-acetyllactosamine (Fig 10B). Products 6’sialyl-*N*-acetyllactosamine and 6’-sialyllactose (the latter using 2 μm enzyme) were obtained in quantitative and 35% yields, respectively. This is in agreement with studies showing the affinity of rat ST6Gal1 (which shares 80% identity with its human homologue) for lactose is 80-times lower than that for *N*-acetyllactosamine.

3’- and 6’- sialyl-*N*-acetyllactosamine and 3’-sialyl-Gal-β-1,3-GalNAc-α-*O*-Bn were produced in mg scale, purified and their identity confirmed by mass spectrometry (S6 Fig).

Degradation of sialylated products was not observed over long periods of incubation with hSTs (22 h), indicating hST3Gal1 and hST6Gal1 either do not possess sialidase activity or this activity is extremely low to be detected during the analyzed reaction times. These results are in agreement with those reported for pST3Gal1 on CMP-Neu5Ac,[16] and for hST6Gal1 on 6’-sialyllactosamine.[34]

## Conclusion

Given their outermost position on glycoconjugates, sialic acids play a key role in in many physiological and pathological events.[2] Unfortunately, the difficult heterologous expression of eukaryotic sialyltransferases limits their availability restricting biochemical and structural studies and hindering their biotechnological application.

We report here the expression of functional human sialyltransferases ST3gal1 and ST6Gal1 in *E*.*coli*. These enzymes contain continuous and discontinuous disulfide bonds and are glycosylated in their native form, with both modifications accounting for difficulties in the production of some eukaryotic sialyltransferases in bacterial systems. We used hST3Gal1 as a model to study the influence of solubility enhancer tags and redox environment in the expression of functional sialyltransferases in bacteria. While the catalytic domain of an *N*-terminal truncated hST3Gal1 was produced only as inclusion bodies, its fusion with MBP resulted in soluble, albeit inactive protein when expressed under reducing conditions. Sialyltransferase activity was only detected upon expression of the MBP-fused protein in an oxidative environment and this activity was increased by co-expression of enzymes assisting the correct pairing of cysteines. Although the role of each disulfide bond in folding and activity of hST3Gal1 remain to be established, an oxidative environment and the presence of enzymes able to correct cysteine mispairing greatly contributed for the expression of active proteins. Yet, a major fraction of soluble fusion enzyme failed to fold into a catalytically active state. Since conditions for correct formation of disulfide bonds were provided, the lack of glycosylation may have favored aggregation and misfolding during overexpression in *E*. *coli*. In fact, the role of glycosylation in folding, stabilization and intracellular traffic of hST3Gal1, hST6Gal1 and other sialyltransferases *in vivo* was already demonstrated. Besides its role as solubility enhancer, it is known that maltose binding protein may function as a “passive chaperone”, which means it may bind and release its partially folded partner in an iterative manner, resulting in spontaneous native folding and avoiding self-aggregation.[35] The fact that a population of MBP-hST3Gal1 was able to reach the active fold, as demonstrated by activity and circular dichroism experiments, indicates MBP can partially fulfill the role of glycosylation in folding. However, expression of higher amounts of active STs may require the use of additional chaperon/foldase systems in combination with a better regulation of the protein expression in order to kinetically compete with the aggregation pathway.

We also showed that mutation of five exposed hydrophobic residues of hST3Gal1 by negatively charged amino acids has a positive influence in protein folding, and marginal activity was recovered even in the absence of a solubility enhancer. In support of our observation, there is evidence of the correlation of exposed negative charges with increased solubility and stabilization during the folding process. While solubility increases as a result of water binding tightly to aspartic and glutamic residues on the protein surface, a native folding seems to be promoted by electrostatic repulsion between nascent polypeptides during translation.[36, 37] Further investigation of the role of the reported mutations in folding thermodynamics may contribute to the design of variants for efficient expression in bacterial systems.

## Methods

### Chemicals

CMP-Neu5Ac disodium salt, benzyl 2-acetamido-2-deoxy-3-*O*-(β-d-galactopyranosyl)-α-d-galactopyranoside (Gal-β-1,3-GalNAc-α-*O*-Bn), *N*-acetyllactosamine, 6'-α-sialyl-*N*-acetyllactosamine sodium salt, 3'-α-sialyl-*N*-acetyllactosamine sodium salt, 6'-sialyllactose sodium salt and 3'-sialyllactose sodium salt were purchased from Carbosynth (Berkshire, UK). Nucleoside Monophosphate Kinase (NMPK) was purchased from Roche (Mannheim, Germany). Lactate dehydrogenase/pyruvate kinase solution (PK/LDH) and IGEPAL-CA-630 were purchased from SIGMA (Steinheim, Germany). Phosphoenolpyruvic acid monopotassium salt (PEP) and NADH were acquired from Alfa Aeasar (Karlsruhe, Germany) and AppliChem (Darmstadt, Germany) respectively. TLC silica gel 60 F_240_ plates were obtained from Merck (Darmstadt, Germany) and electrophoresis reagents from Merck and BDH-Prolabo (Darmstadt, Germany).

### Construction and cloning of wild type and mutant sialyltransferases

DNA encoding human ST3Gal1 [Uniprot: Q11201] and ST6Gal1 [Uniprot: P15907] with optimized codon usage for expression in *E*. *coli* cells were synthesized starting from Glu45 (Δ44) and Leu48 (Δ47) respectively (Mr. Gene, Regensburg, Germany, now renamed as GeneArt, Life technologies). Genes contain *NdeI*-*EcoRI* restriction sequences at 3’ and 5’ ends respectively and were cloned into the vector pMAL-c5X (New England BioLabs, Ipswich, MA). ST3Gal1-Δ44 was also cloned into pET28b+ and in pLgals1-Tev between restriction sites *NdeI*-*HindIII* and *EcoRI*-*HindIII* respectively. pLgals1-Tev (a gift from Dr. Qasba, SAIC-Frederick) is a vector derived from pET23a that includes the sequence for human galectin-1, followed by the Tev protease cleavage site, a 6x His-coding sequence, and a multi-cloning site.[22]

hST3Gal1-Δ44 gene was used as a template for amplification of an *N*-terminal variants with five *N*-terminal residues added to generate Δ39 (starting at Lys40). In turn, this variant was used as a template for amplification of an *N*-terminal Δ34 variants with additional 5 amino acids added (starting at Thr35). The three *N*-terminal variants were cloned into pETM-50 and pETM-80 vectors between restriction sites *NcoI*-*Acc65I*. Constructs cloned into vectors pETM-50 and pETM-80 (https://www.embl.de/pepcore/pepcore_services/cloning/choice_vector/ecoli/embl/popup_emblvectors/) are secreted into the periplasm. Primers used for PCR are shown in S1 Table.

### Periplasmic expression of hST3Gal1

pETM-50 and pETM-80 plasmids containing hST3Gal1 genes were transformed in BL21(DE3) and selected on LB plates with kanamycin (50 μg/mL final concentration). One colony was used to inoculate 5 mL LB medium and overnight cultures were used to inoculate 50 mL LB broth. Cultures were grown at 37°C and 200 rpm shaking until an OD_600_ of 0.55–0.65 was reached. Temperature was adjusted to either to 18 or 30°C for STs expression, which was induced by adding IPTG to a final concentration of 0.1 mm. Cultures were incubated over 4 or 22 hours and cells harvested by centrifugation (6500 x g, 15 min). Additives i.e. sucrose (0.5 m), reduced glutathione (5 mm), ethanol (1% v/v), arginine (0.4 m) and sorbitol (0.4 m) were added at the beginning of the culture or at induction time to enhance solubility of fusion proteins.

For extraction of the periplasmic fraction, pellets were resuspended in 20 ml of 30 mm Tris-HCl, pH 8.0, with 20% sucrose and 1mm EDTA. Following 10 minutes incubation at room temperature with gentle shaking, pellets were centrifuged at 8000 x g at 4°C for 20 minutes, supernatant was removed and pellets were resuspended in 20 mL ice-cold 5 mm MgSO_4_. Samples were incubated for 20 minutes at 4°C with moderate shaking followed by centrifugation. Supernatant was recovered and analyzed for expression and activity.

### Protein supercharging

A three dimensional model of human ST3Gal1 was generated by the SWISS-MODEL server using porcine ST3Gal1 as template [PDB: 2WNB]. The model was used to analyze solvent accessibility of ST3Gal1 residues and to detect hydrophobic patches. Five solvent-accessible residues involved in short hydrophobic patches were chosen for mutagenesis and exchanged by either aspartic or glutamic acid. ST3Gal1 gene cloned into the vector pMAL-c5x was used as a template to generate a quintuple variant bearing mutations L70D, L92E, A175E, T225E and A326E by using the Quikchange lightning multi-site-directed mutagenesis kit (Stratagene, CA, USA) according to manufacturer’s recommendations. Primer sequences are shown in S1 Table.

### Expression in E. coli BL21, SHuffle and Origami

pMAL constructs were expressed as *N*-terminus MBP fusion proteins carrying a *C*-terminal His tag and selected on medium containing ampicillin (200 μg/ml final concentration). pET28b+ constructs were expressed as *N*-terminus His tagged enzymes and selected on kanamycin (30 μg/ml final concentration). The pLgals1 construct is the fusion of an *N*-terminal human galectin-1 with hST3gal1 and was selected with 100 μg/ml ampicillin. Plasmids were transformed for expression in *E*. *coli* BL21 (New England BioLabs), SHuffle T7 Express (New England BioLabs) and Origami 2 DE3 (Novagen, Darmstadt, Germany) and plated onto LB agar plates containing the appropriate antibiotic. Tetracycline (12.5 μg/ml final concentration) was also included for selection of the gor mutation in Origami. One colony was used to inoculate 5 mL LB medium and overnight cultures were used to inoculate 500 mL LB broth. Cultures were grown at 30°C and 200 rpm shaking until an OD_600_ of 0.55–0.65 was reached. Temperature was dropped to 17°C for expression of hST3Gal1 and hST6Gal1. Sialyltransferase expression was induced by adding IPTG to a final concentration of 0.1 mm. Cultures were incubated over 22 hours and cells harvested by centrifugation (6500 x g, 15 min) and washed. Pellets were resuspended in 7 mL lysis buffer (25 mm NaH_2_PO_4_, 25 mm Na_2_HPO_4_, 300 mm NaCl and 20 mm imidazole, pH 8) and frozen at -20°C.

### Pre- and co-expression of sialyltransferases with chaperon/foldases

Plasmids bearing hST3Gal1 and hST6Gal1 genes were transformed in Origami2 DE3 already carrying either plasmids pMJS9 or pMJS10 (kindly provided by Prof. Ruddock, University Oulu, Finland). pMJS9 and pMJS10 carry encoding genes for enzymes Erv1/PDI (Sulfhydryl oxidase/Protein disulfide isomerase) and Erv1p/DsbC (Sulfhydryl oxidase/Disulfide isomerase C) respectively and are selected on medium containing chloramphenicol (30 μg/ml final concentration). Five mL overnight cultures were used to inoculate 500 mL LB broth in 2 L baffled shake flask containing the appropriate antibiotic combination. Cells were grown at 30°C and 200 rpm shaking and expression of Erv1/PDI and Erv1p/DsbC was induced at an optical density of around 0.4 by adding L-(+)-arabinose to a final concentration of 0.5%. Temperature was dropped to 17°C for expression of hST3Gal1 and hST6Gal1when cultures reached an optical density of 0.55–0.65. Sialyltransferases expression was induced by adding IPTG to a final concentration of 0.1 mm. Cultures were incubated over 22 h and cells harvested by centrifugation (6500 x g, 15 min) and washed. Pellets were resuspended in 7 mL lysis buffer (25 mm NaH_2_PO_4_, 25 mm Na_2_HPO_4_, 300 mm NaCl and 20 mm imidazole, pH 8) and frozen at -20°C.

### Purification

Enzymes for biochemical and structural characterization were purified by IMAC. Briefly, frozen cells were thawed at room temperature and glycerol was added to a final concentration of 10% (v/v) before cell lysis by sonication on ice. Lysates were centrifuged at 4°C and 16,000 x g for 30 min and supernatants filtrated through a 0.2 μm filter. Protein was quantified by the Bradford method and loaded at same protein concentration (4 mg/mL) onto gravity Ni-NTA columns (0.8 mL resin each) previously equilibrated with lysis buffer. After 2 h incubation at 4°C, supernatants were allowed to flow through and columns were washed with 20 mL of lysis buffer. Sialyltransferases were eluted in a single step with 2.5 mL elution buffer (25 mm NaH_2_PO_4_, 25 mm Na_2_HPO_4_, 300 mm NaCl and 250 mm imidazole, pH 8). Purified proteins were buffer exchanged (50 mm HEPES, pH 7.5, 200 mm NaCl and 20% Glycerol) and concentrated on Vivaspin-500 ultrafiltration tubes with a MWCO of 50,000 Da (Goettingen, Germany).

Human sialyltransferases bearing maltose binding domain at the *N*-terminus were also purified by affinity chromatography using an amylose resin according to manufacturer’s recommendations (New England BioLabs).

### Size-exclusion chromatography (SEC)

*E*. *coli* cleared cell lysates containing recombinant hST3Gal1 and purified sialyltransferases were analyzed by SEC. Samples were prepared in 50 mm PBS buffer, pH 7.4 and 10% glycerol. 0.3 mL of cell lysates at a final concentration of 4 mg/mL and purified proteins at a final concentration of 0.2 mg/mL were applied to a HiLoad 16/600 Superdex 200 prep grade column (GE Healthcare, Germany) coupled to an Äkta purification system (GE Healthcare, Germany) and eluted with PBS buffer, pH 7.4 at 0.8 mL min^-1^ at 4°C. Elution fractions were analyzed by SDS-PAGE. Molecular weight of MBP-fused and MBP-cleaved sialyltransferases was determined by comparing its elution volume with that of known SEC protein standards (SIGMA, Steinheim, Germany).

### Proteolytic cleavage of sialyltransferase from MBP

Purified fusion proteins were incubated with Factor Xa protease, which recognizes the peptide sequence Ile-(Glu or Asp)-Gly-Arg and cleaves the fusion after the arginine residue. Reactions containing 20 mm Tris-HCl, pH 8.0, 2 mm CaCl_2_, fusion protein (1–2 mg/mL) and Factor Xa protease at a final concentration of 3 μg/mL were incubated for 72 h at 10°C. Afterwards cleaved STs, which have a C-terminal His-tag were purified by IMAC as described above and concentrated on Vivaspin-500 ultrafiltration tubes with a MWCO of 10,000 Da.

### Protein quantification

Protein concentration of cleared lysates was determined by the Bradford method using bovine serum albumin as standard. To determine the concentration of purified proteins, absorbance was measured at 280 nm and sialyltransferases’ extinction coefficient was used for protein quantification.

### Electrophoresis

Expression of sialyltransferases in different systems and purification were analyzed by SDS-PAGE applying crude lysates, soluble fractions and purified proteins onto 12% acrylamide gels.

### Continuous coupled activity assay

Initial rates were determined using the continuous spectrophotometric assay in which UDP is coupled to NADH oxidation via pyruvate kinase and lactate dehydrogenase.[38] UDP is produced by action of enzyme NMPK from ATP and CMP released in the reaction with sialyltransferases. Kinetic parameters were obtained at 37°C using a GENios plate reader (Tecan, Switzerland) by varying the concentration of acceptor (Gal-β-1,3-GalNAc-α-*O*-Bn) from 5.0 μm to 3.0 mm at 700 μm of donor (CMP-Neu5Ac), or varying the concentration of donor from 10 μm to 1.2 mm at 1.0 mm of acceptor. Reactions were carried out in 100 μL on 384 well plates containing: 50 mm HEPES, pH 7,4, 0.7 mm PEP, 0.24 or 0.29 mm NADH, 2 mm ATP, 50 mm KCl, 10 mm MnCl_2_, 10 mm MgCl_2_, 1 g/L BSA, 15 mU of NMPK, 8 U of PK, 12 U of LDH and varying concentrations of donor and acceptor. Well plates were centrifuged at 1000 x g to spin down samples for 30 s and reactions were incubated at 37°C for 22 min to deplete CMP present in donor solution and NDPs from ATP and NMPK solutions.[32] This incubation time is required to obtain the spontaneous hydrolysis rate of CMP-Neu5Ac. After this period of incubation, 2 μL of sialyltransferase solution was added, the well plate was centrifuged at 1000 x g for 30 s and the change in absorbance was recorded for 40 min while incubating at 37°C. An extinction coefficient for NADH of 6220 m^-1^ cm^-1^and a path length of 0.82 cm was used in calculations for initial rates. K_M_ and k_cat_ values were calculated by fitting the data to the Michaelis-Menten equation using non-linear curve in OriginPro (OriginLab).

One unit is defined as the amount of protein that transfers 1 μmol of Neu5Ac from CMP- Neu5Ac to either Gal-β-1,3-GalNAc-α-*O*-Bn (in the case of ST3Gal1) or *N*-acetyllactosamine (for ST6Gal1) per min at 37°C, pH 7.4.

400 μm Gal-β-1,3-GalNAc-α-*O*-Bn and 700 μm CMP-Neu5Ac were used to determine specific activity of hST3Gall recovered by IMAC purification from different expression systems.

### Circular dichroism spectroscopy

CD spectra of MBP-fused and MBP-cleaved sialyltransferases were recorded with a JASCO model J-810 spectropolarimeter. Measurements were performed using protein concentration of 0.8–1 μm in 50 mm Soerenson buffer, pH 6.5 at 20°C. CD spectra were recorded over a range of 190 to 280 nm with a response of 0.5 at 0.1 nm and a scanning speed of 100 nm/min. 10 accumulations were averaged to reduce noise. Buffer blank spectrum was obtained at identical conditions and subtracted.

### Synthesis of sialyl-products and analysis of sialyl-products by HPAEC-PAD

Reaction containing 0.7 mm CMP-Neu5Ac, 0.4 mm Gal-β-1,3-GalNAc-α-*O*-Bn and cleared cell lysates containing expressed sialyltransferases at a final concentration of 50 μg mL^-1^ total extract protein were prepared in 20.0 mM MOPS, pH 7.5 in 80.0 μL final volumes. Reactions were incubated at 37°C and samples were taken over the reaction time of 2 h and analyzed by High-performance Anion Exchange Chromatography (HPAEC) with a Dionex ICS-5000+ SP system utilizing a Carbopac PA10 column (250 x 2 mm) at a flow of 250 μL min^-1^. Eluents were 100 mm NaOH (A), 100mm NaOH, 1 m NaOAc (B), and 250 mm NaOH (C). Acceptor molecules and sialosides were resolved using a multistep gradient programmed as follows: 0–5 min 100% A, 5–20 min 0–20% B, 20–21 min 20–45% B, 21–28 min 45% B, 28-to 35 min 100% C, 35–45 min 100% A. Acceptor consumption and sialosides synthesis were calculated using appropriate standards (sialosides from Carbosynth).

For production of sialosides, reaction containing 4.50 mm CMP-Neu5Ac, 3.0 mm acceptor (Gal-β-1,3-GalNAc-α-*O*-Bn, lactose or *N*-acetyllactosamine) and sialyltransferases at a final concentration of 1.0 or 2.0 μm were prepared in 20.0 mM MOPS, pH 7.5 in 40.0 μL final volumes. Reactions were incubated at 37°C and samples withdrawn over the reaction time of 22 h and analyzed by thin-layer chromatography (TLC) and HPAEC-PAD.

### TLC

Reaction samples were spotted on TLC plates that were afterwards developed in a system containing water, isopropanol and ethyl acetate (2:3:5). After four ascends, *N*-(1-naphthyl) ethylenediamine in methanol/concentrated sulfuric acid (97/3, v/v) was used for sugar visualization.

### Sialosides mass spectrometry analysis

Reactions containing 8 mm donor CMP-Neu5Ac, 6 mm acceptor (Gal-β-1,3-GalNAc-α-*O*-Bn, lactose or *N*-acetyllactosamine) and 1 μM sialyltransferase in 20 mm MOPS, pH 7.5 were incubated at 37°C for 20 h. Sialosides were purified by size exclusion Bio-Gel P-2, using water as eluent, followed by silica gel chromatography using water, isopropanol and ethyl acetate as eluents (2:3:5 ratio).

Samples were dried and dissolved in methanol for electrospray ionization mass spectrometry analysis.

## Supporting Information

S1 Fig SDS-PAGE of MBP-hST3Gal1 expression in different systems. A) Cleared lysates before incubation with IMAC resin, and B) unbound protein after IMAC purification (flow-through fraction).
(TIF)

S2 Fig Effect of additives on the recovery of MBP-hST3Gal1 by IMAC. Cleared lysates containing the same protein concentration and different additives (A) were loaded onto IMAC columns. After incubation the flow-through (B) and elution (C) fractions were collected and analyzed by SDS-PAGE.
(TIF)

S3 Fig Specific activity of MBP-hST3Gal1-wt and its variant produced in different expression systems. Activities reported are the average of at least 5 culture batches.
(TIF)

S4 Fig Expression and activity of His- and MBP-tagged sialyltransferases hST3Gal1-wt and hST3Gal1-5x. Activity of BL21 and Origami cleared extracts containing different STs constructs was detected by HPAEC-PAD after 2 h incubation with 0.4 mm Gal-β-1,3-GalNAc-α-*O*-Bn and 0.7 mm CMP-Neu5Ac. Synthesis of 3’-sialyl-Gal-β-1,3-GalNAc-α-*O*-Bn is shown. Retention time shift observed for some samples is the result of small differences in HPAEC-PAD eluents. SDS-PAGE is composed of 3 gels, which is indicated by vertical black lines. His- and MBP-wt: His-hST3Gal1-wt and MBP-hST3Gal1-wt respectively. His- and MBP-5x: His-hST3Gal1-5x and MBP-hST3Gal1-5x variants respectively. ORI: Origami2 (DE3).
(TIF)

S5 Fig Kinetics behavior of MBP-fused and MBP-cleaved sialyltransferases hST3Gal1-wt and hST3Gal1-5x. Kinetic parameters were obtained at 37°C, varying the concentration of the acceptor (Gal-β-1,3-GalNAc-α-*O*-Bn) from 5.0 μm to 3.0 mm at 700 μm of the donor (CMP-Neu5Ac), or varying the concentration of the donor from 10 μm to 1.2 mm at 1.0 mm of the acceptor.
(TIF)

S6 Fig Mass spectra of 6’- sialyl-*N*-acetyllactosamine and 3’- sialyl- Gal-β-1,3-GalNAc-α-*O*-Bn. A) ESI (negative) spectrum of 6’- sialyl-*N*-acetyllactosamine: calculated mass for C_25_H_41_N_2_O_19_ = 673.23090; experimental mass = 673.23100 (error = 0.15 ppm). B) ESI (positive) spectrum of 3’-sialyl-Gal-β-1,3-GalNAc-α-*O*-Bn: calculated mass for C_32_H_48_N_2_O_19_Na = 787.27435; experimental mass = 787.27594 (error = -2.02 ppm). (TIF)

S1 Table Sequence of primers used for the construct of *N*-terminal variants of hST3Gal1 and the quintuple mutant hST3Gal1-5x. (DOCX)

## Abbreviations

MBP: Maltose binding protein
hST3Gal1: Human ST3Gal1
hST6Gal1: Human ST6Gal1
HPAEC-PAD: High Performance Anion Exchange Chromatography with Pulsed Amperometric Detection
TLC: Thin-layer chromatography
IMAC: immobilized metal ion affinity chromatography
SEC: Size-exclusion chromatography
IPTG: Isopropyl β-D-1-thiogalactopyranoside
