# Supplementary figures and images for: Expression of Functional Human Sialyltransferases ST3Gal1 and ST6Gal1 in Escherichia coli
Source: PLoS One. 2016 May 11;11(5):e0155410. doi: 10.1371/journal.pone.0155410 (PMC4864186; doi:10.1371/journal.pone.0155410)

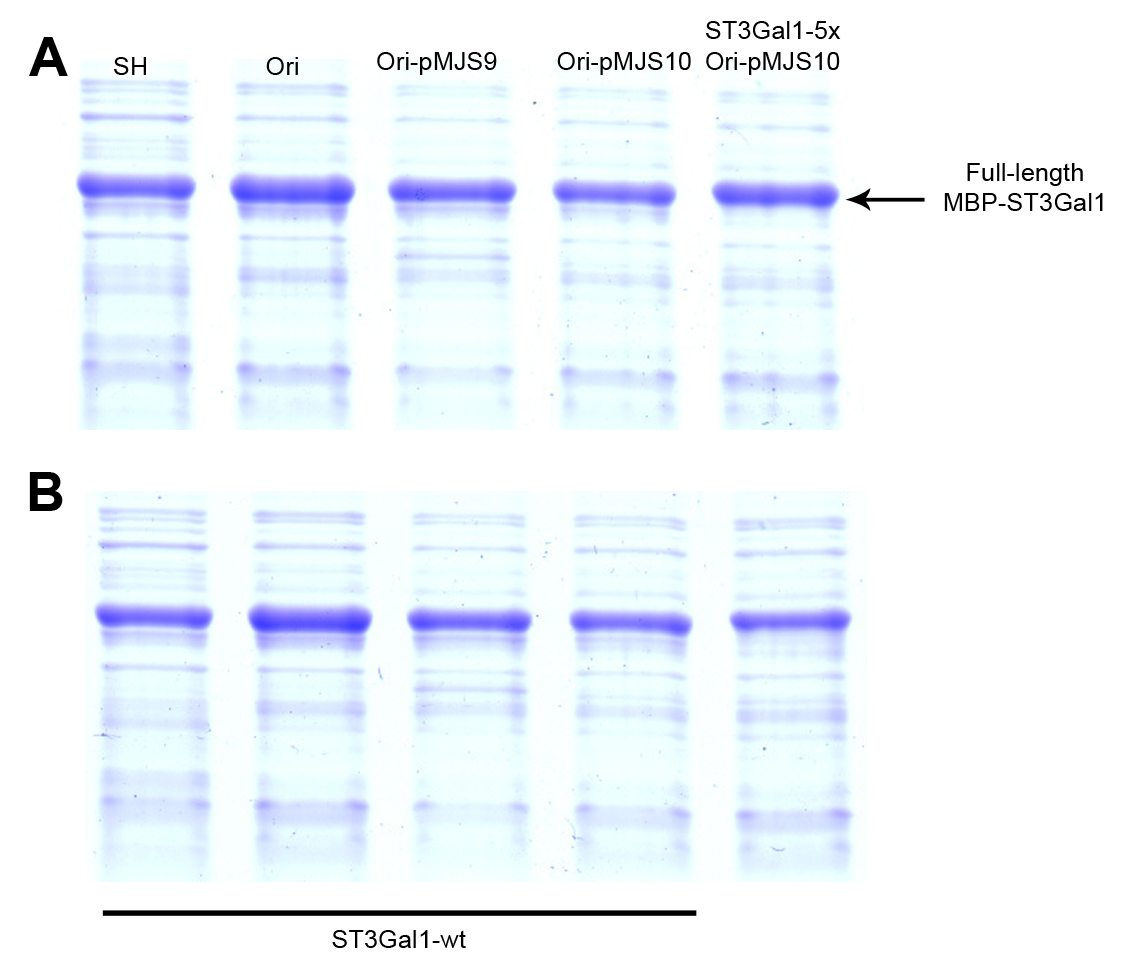

Supplement: S1 Fig — A) Cleared lysates before incubation with IMAC resin, and B) unbound protein after IMAC purification (flow-through fraction). (TIF) [file pone.0155410.s001.tif]

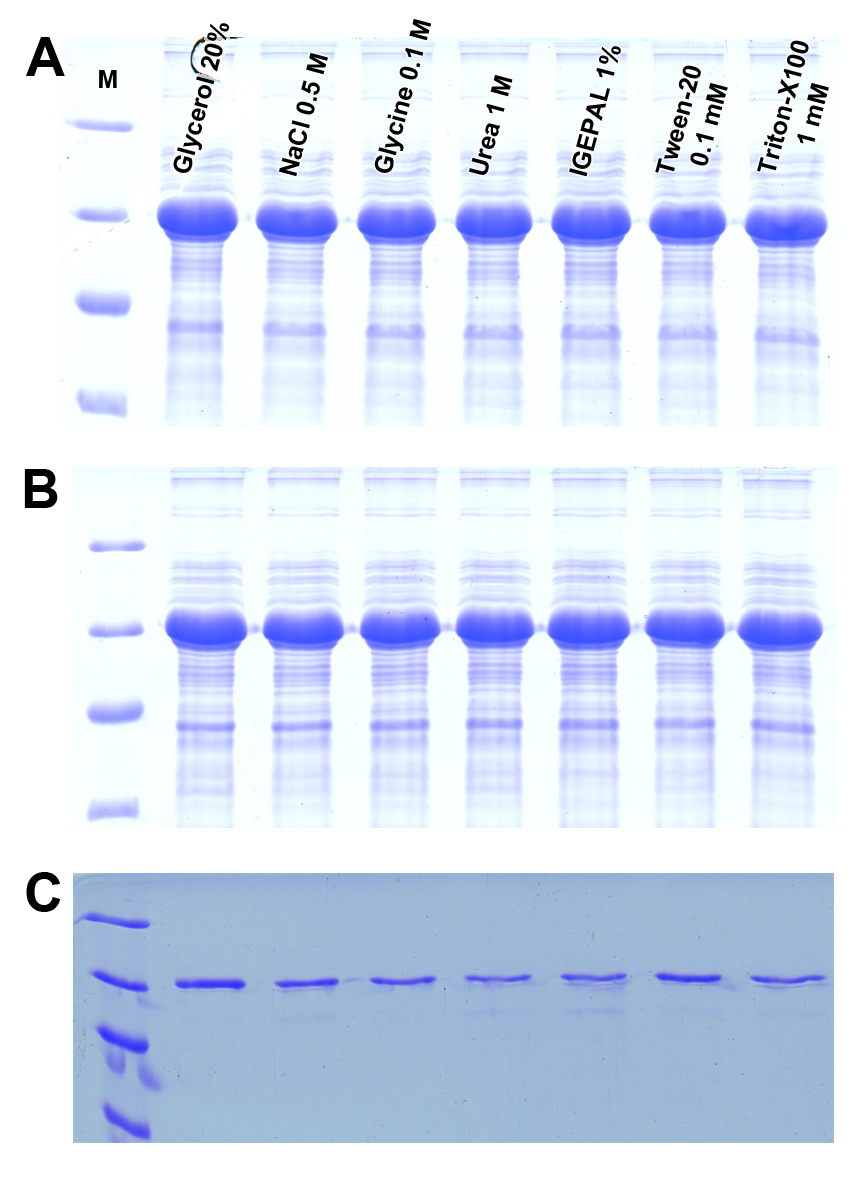

Supplement: S2 Fig — Cleared lysates containing the same protein concentration and different additives (A) were loaded onto IMAC columns. After incubation the flow-through (B) and elution (C) fractions were collected and analyzed by SDS-PAGE. (TIF) [file pone.0155410.s002.tif]

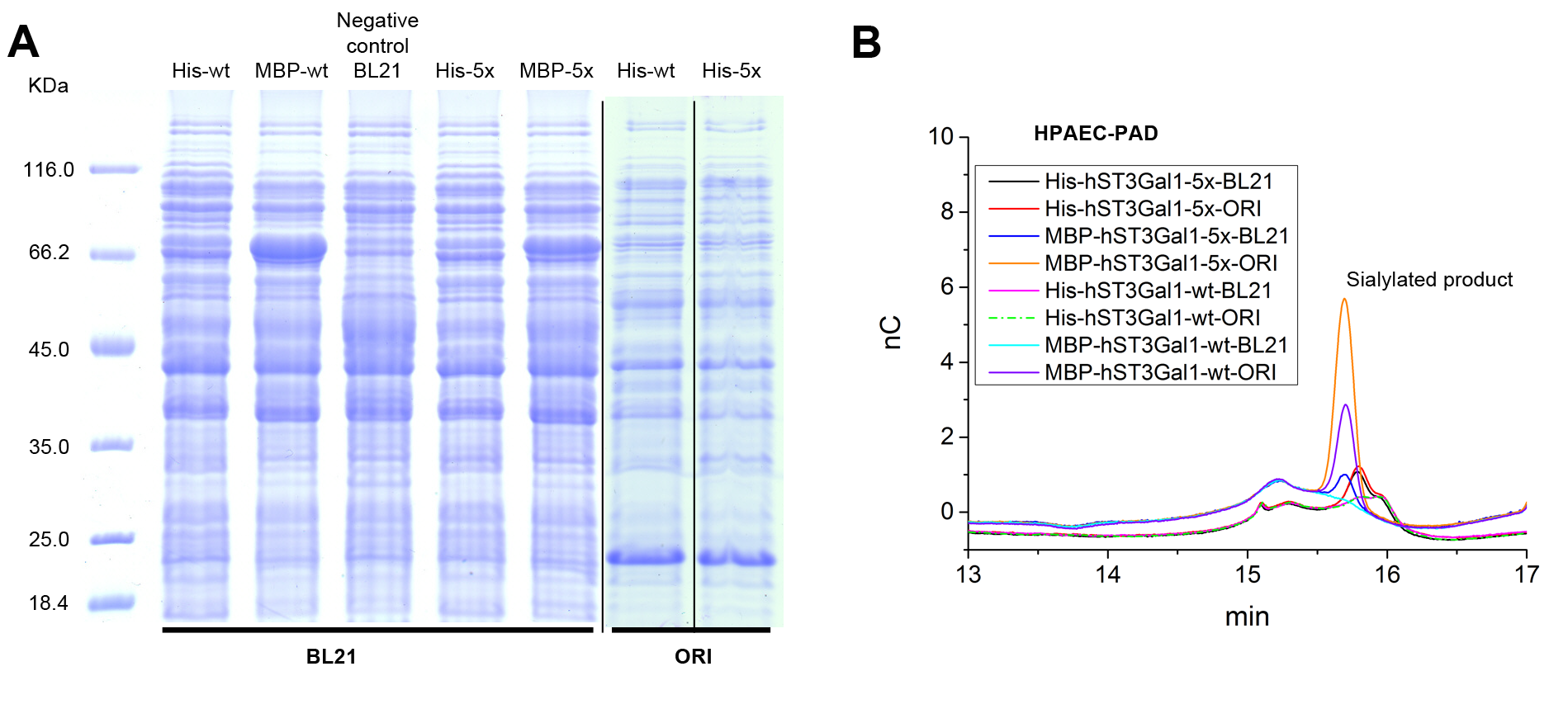

Supplement: S4 Fig — Activity of BL21 and Origami cleared extracts containing different STs constructs was detected by HPAEC-PAD after 2 h incubation with 0.4 mm Gal-β-1,3-GalNAc-α-O-Bn and 0.7 mm CMP-Neu5Ac. Synthesis of 3’-sialyl-Gal-β-1,3-GalNAc-α-O-Bn is shown. Retention time shift observed for some samples is the result of small differences in HPAEC-PAD eluents. SDS-PAGE is composed of 3 gels, which is indicated by vertical black lines. His- and MBP-wt: His-hST3Gal1-wt and MBP-hST3Gal1-wt respectively. His- and MBP-5x: His-hST3Gal1-5x and MBP-hST3Gal1-5x variants respectively. ORI: Origami2 (DE3). (TIF) [file pone.0155410.s004.tif]

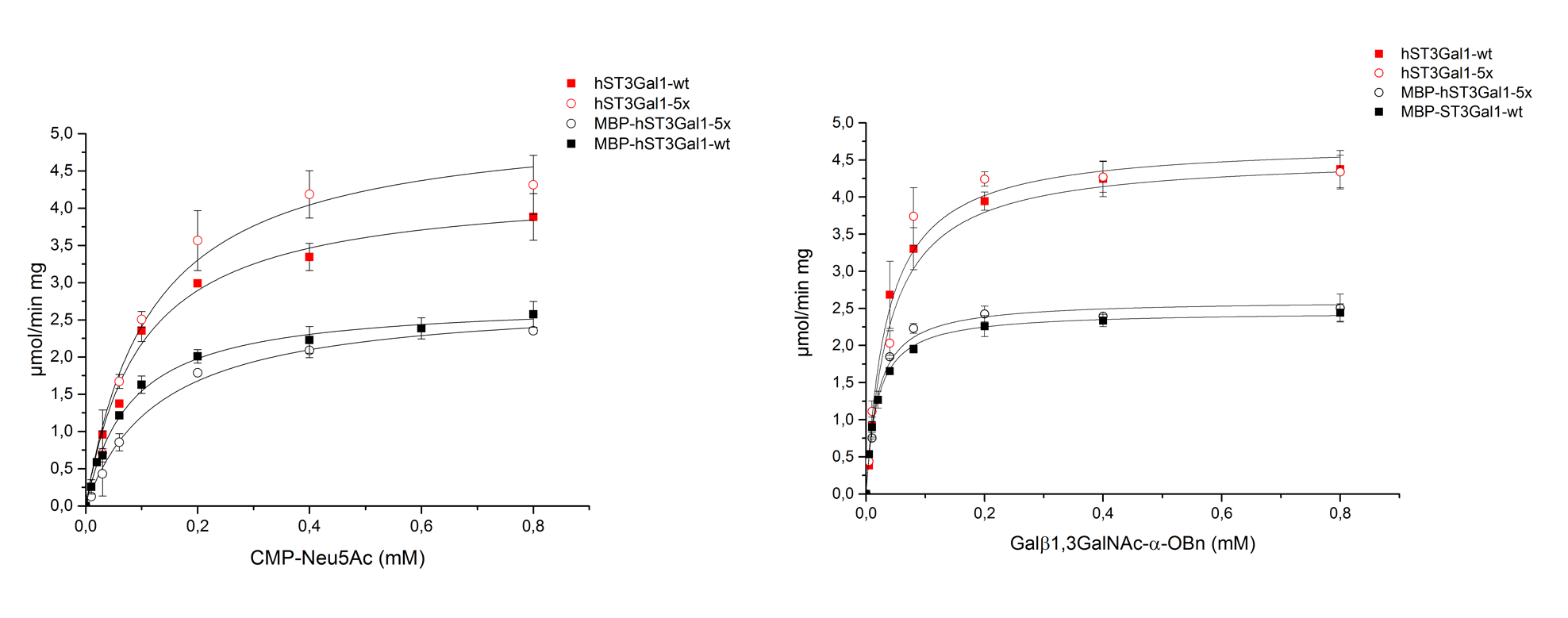

Supplement: S5 Fig — Kinetic parameters were obtained at 37°C, varying the concentration of the acceptor (Gal-β-1,3-GalNAc-α-O-Bn) from 5.0 μm to 3.0 mm at 700 μm of the donor (CMP-Neu5Ac), or varying the concentration of the donor from 10 μm to 1.2 mm at 1.0 mm of the acceptor. (TIF) [file pone.0155410.s005.tif]

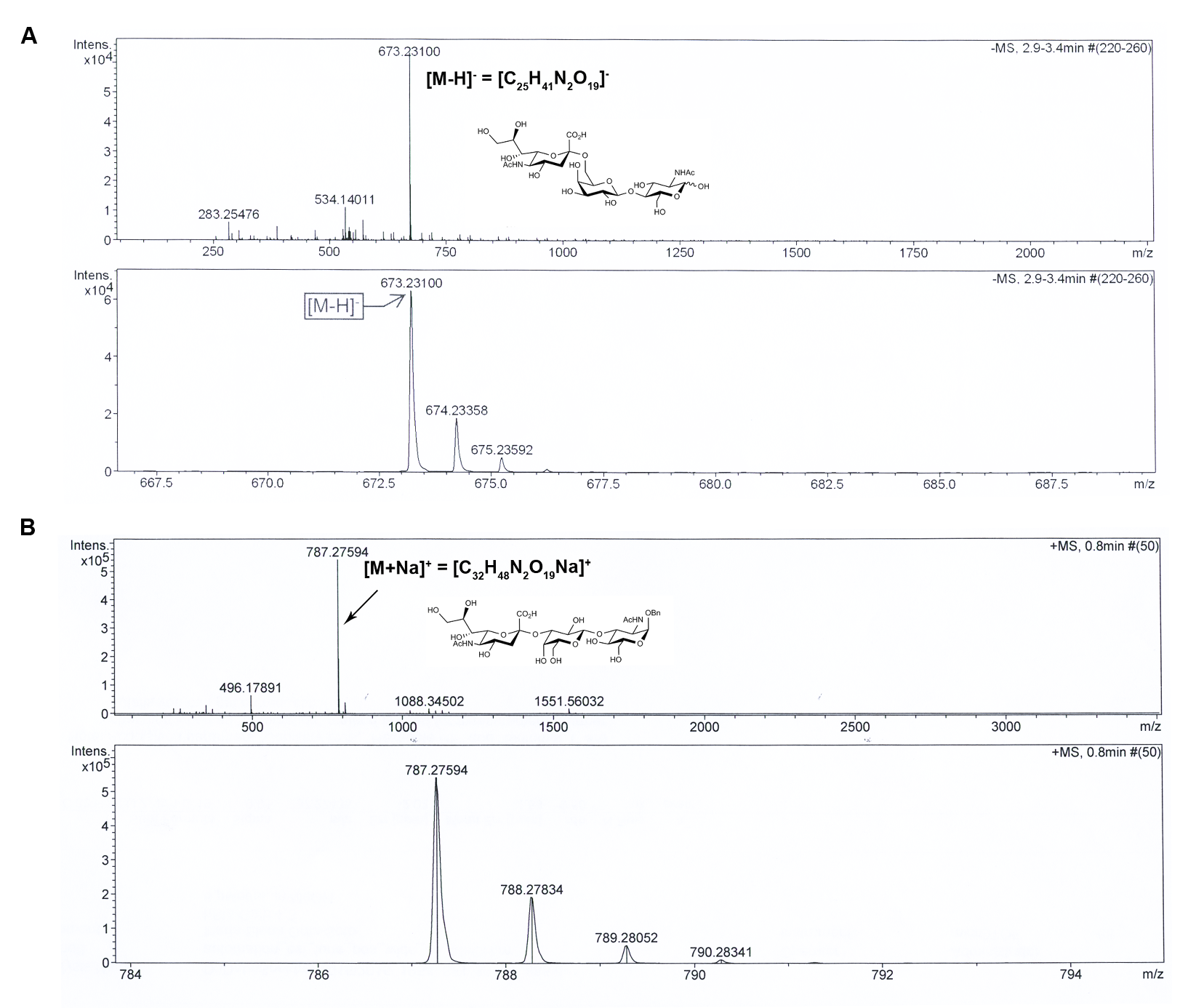

Supplement: S6 Fig — (TIF) [file pone.0155410.s006.tif]
