# Supplementary material for: Expression of Functional Human Sialyltransferases ST3Gal1 and ST6Gal1 in Escherichia coli
Source: PLoS One. 2016 May 11;11(5):e0155410. doi: 10.1371/journal.pone.0155410 (PMC4864186; doi:10.1371/journal.pone.0155410)
Supplement: S1 Table — (DOCX) [file pone.0155410.s007.docx]

| Primer | Sequence |
| --- | --- |
| NcoI-hST3gal1(Δ34)-Fw | GGCCATGGAAACCACCTGGTTCCCGAAACAGATGGTTCTGGAAC |
| NcoI-hST3gal1(Δ39)-Fw | GGCCATGGAAAAACAGATGGTTCTGGAACTGAGCGAGAACCTG |
| NcoI-hST3gal1(Δ44)-Fw | GGCCATGGAACTGAGCGAGAACCTGAAAC |
| KpnI-hST3gal1-Rv | GTTGGTACCTCAGCGGCCTTTGAAAATAC |
| L70D-Fw | GTATTGGTCAGCGTAAAGATTCAGCCTGGTTTGATG |
| L70D-Rv | CATCAAACCAGGCTGAATCTTTACGCTGACCAATAC |
| L92E-Fw | GACTGCTCAAAATGCTGAACTGGAGGATGACACC |
| L92E-Rv | GGTGTCATCCTCCAGTTCAGCATTTTGAGCAGTC |
| A175E-Fw | GAATAAAGCCCCGACTGAAGGATTCGAAGCTGATG |
| A175E-Rv | CATCAGCTTCGAATCCTTCAGTCGGGGCTTTATTC |
| T225E-Fw | GTGCTATCACTACTGGCGAAATCTCACACACTTATATC |
| T225E-Rv | GATATAAGTGTGTGAGATTTCGCCAGTAGTGATAGCAC |
| A326E-Fw | GAGAGCAATGTTACCGAAACACTGGCAAGCATC |
| A326E-Rv | GATGCTTGCCAGTGTTTCGGTAACATTGCTCTC |

Table S1. Sequence of primers used to construct the N-terminal variants of hST3Gal1and its quintuple mutant.
